# Emergency department interventions and their effect on subsequent healthcare resource use after discharge: an overview of systematic reviews

**DOI:** 10.1186/s13049-025-01377-4

**Published:** 2025-05-01

**Authors:** Tom Roberts, Callum Taylor, Edward Carlton, Mathew Booker, Sarah Voss, Nicola Trevett, Daniel Wattley, Jonathan Benger

**Affiliations:** 1 Royal College of Emergency Medicine, London, UK; 2 North Bristol NHS Trust, Bristol, UK; 3 University Hospitals Bristol NHS Foundation Trust, Bristol, UK; 4 University of the West of England, Bristol, UK; 5 University of Bristol, Bristol, UK

## Abstract

**Background:**

Due to the worldwide pressures on Emergency Departments (EDs), there is a focus on ED interventions to alleviate pressure. Ensuring interventions do not inadvertently impact upon other healthcare sectors is an important outcome. This overview of systematic reviews aimed to evaluate the impact of ED based interventions on subsequent healthcare resource use after ED discharge.

**Methods:**

An overview of systematic reviews was conducted in accordance with the Cochrane Collaboration. Search criteria were devised using the PRESS standard and duplicate screening and extraction conducted for one third of systematic reviews. A primary study matrix was designed to reduce the impact of duplicate primary studies. Data was extracted in the form presented in the underlying review.

**Results:**

After removal of overlapping primary studies, 38 systematic reviews and 213 primary studies were included. Overall confidence in the reviews was high in 12, moderate in seven, low in nine and critically low in 10 reviews. In the 38 reviews, 30 different intervention-population-resource use combinations were analysed. ED based interventions decreased subsequent healthcare resource use in 23.3% (n = 7/30) of the intervention-population-resource use combinations and had no effect in 40% (n = 12/30). The most common resource use reported was ED Revisit. The most common follow-up length from ED discharge was 12 months (n = 52/216), followed by the combined group of one month (n = 44/216).

**Conclusions:**

ED based interventions decrease subsequent healthcare resource use in a fifth of population-intervention-resource use combinations. Future research should produce a standardised set of outcome measures for subsequent healthcare resource use.

**Supplementary Information:**

The online version contains supplementary material available at 10.1186/s13049-025-01377-4.

## Background

Worldwide pressures across the Emergency Care system are unprecedented [1–3]. In the United Kingdom (UK), healthcare pressures extend to the primary care system [4], emergency medical service (EMS) system [5] and elective care [6].

To date, policy and research efforts to combat ED pressures has focused on interventions designed to re-direct patients away from EDs, reduce ED use or improve ED flow, but there is little evidence to support these interventions [7–9]. Pre-hospital and ED interventions do not decrease the proportion of patients transferred to hospital [7], evidence of the effectiveness of interventions to reduce ED use remains insufficient [8] and the evidence of interventions designed to improve patient flow is weak [9]. It is therefore important to understand the resource implications of these interventions on other sectors of healthcare.

A key outcome measure, infrequently evaluated, is subsequent healthcare resource use after discharge from the ED. Interventions that increase or decrease subsequent healthcare resource use will have systems, resource and patient impacts [10]. Understanding the full impact of ED interventions will ensure the appropriate allocation of limited resources to produce a net health system benefit. Therefore, this overview of systematic reviews, aims to evaluate ED based interventions which report subsequent healthcare resource use as an outcome for interventions.

The four objectives are to (1) identify systematic reviews which report subsequent healthcare resource use as an outcome for interventions designed for ED patients; (2) evaluate interventions that been shown to decrease subsequent healthcare resource use versus interventions that have no effect; (3) identify the theoretical concepts that underpin interventions that are effective; (4) to analyse the variability in definitions of subsequent healthcare resource use in respect to resources and time elapsed from ED discharge.

## Methods

### Study design

This was an overview of systematic reviews and was conducted according to guidance outlined by the Cochrane Collaboration for overviews [11]. It has been reported as per the recommendations in Box V.5.b of the Cochrane guidance [11]. All references to systematic reviews, will use the term ‘review’. The protocol was registered at Prospero (ID = CRD 42021230846).

### Criteria for selecting reviews for inclusion

#### Types of reviews

Reviews and meta-analyses of primary studies (randomised controlled trials (RCT) and/or non-randomised) which evaluated ED based interventions and reported subsequent healthcare resource use as an outcome were included. A review was defined by the five criteria defined by Cochrane [12].

#### Types of participants

Reviews were included if they contained primary studies with an intervention based in the ED that targeted adults (> 18 years). Interventions could focus on any target condition or symptom, ED population or ED process.

#### Types of interventions

Interventions were excluded if based on biomarker blood tests only. This was done to avoid the volume of biomarker diagnostic studies biasing the sample of reviews. Any other review reporting an intervention within the ED that reports subsequent healthcare resource use as a primary, composite or secondary outcome were included.

#### Types of outcome measures

Subsequent healthcare resource use was the outcome measure. The resource use had to be linked to the index ED attendance and a time interval of 12 months from discharge was used. Resource use was divided into the following six categories:

- Attendance to Primary Care/Family Clinician
- Re-attendance to the ED
- Referrals to secondary or tertiary speciality clinic hospital
- Referrals to community clinics
- Contact to telephone triage services (e.g., NHS 111 in the UK)
- Contact to Emergency Medical Services (EMS)

Other outcome measures not described a priori were included if they constituted healthcare-associated resource use post ED discharge. The description of the healthcare resource use was extracted in the format reported in the included review.

#### Search methods for identification of reviews

The search was derived using the PRESS strategy [13], with input from two independent medical librarians and the review team. The search criteria are specified in the online supplement-1. Five databases were searched, Medline, EMBASE, PsycINFO, Cumulative Index to Nursing and Allied Health Literature (CINAHL) and the CENTRAL trials registry of the Cochrane Collaboration. The search was limited to the English language. The reference lists of included reviews were scanned to identify any further reviews for inclusion.

### Data collection and analysis

The search results were uploaded to Covidence, a review management software [14]. Two review authors independently screened titles, abstracts and full texts for inclusion (TR screened all, NT and DW provided independent review). Data extraction of key variables and quality assessments were performed in duplicate for a third of titles (performed by TR and CT). At this time, an inter-rater agreement (κ statistic), was assessed to allow for solo data extraction [15]. Any disagreements between reviewers were resolved with discussion between reviewers, if disagreements remained these were resolved by an independent arbitrator (EC).

### Quality of included reviews

Each review was assessed using the ‘A MeaSurement Tool to Assess systematic Reviews (AMSTAR-2)’ checklist and reported narratively in the results. Each domain and a quality rating of ‘High’, ‘Moderate’, ‘Low’ or ‘Critically low’ are reported [16]. Only ‘High’ or ‘Moderate’ quality reviews are presented in the text. ‘Low’ and ‘Critically Low’ reviews are presented in data tables for reference. As above, AMSTAR-2 ratings were performed in duplicate for a third of titles (TR and CT), the remainder calculated by TR, after the calculation of a suitably high inter-rater agreement (κ statistic).

### Risk of bias of primary studies included in reviews

As outlined in the Cochrane guidance, the risk of bias (RoB) of primary studies from each selected review was extracted directly and was reported narratively, as per Bialey et al. and Foisy et al. [17, 18]. Where a RoB was not reported, a RoB assessment for primary studies was not conducted.

### Quality of evidence in included reviews

Reported ‘The Grading of Recommendations Assessment, Development and Evaluation’ (GRADE) ratings of each outcome in the review were extracted and reported narratively. Any other quality assessments will be reported narratively in the results. If GRADE rating or quality assessment was not done, a new assessment was not conducted.

### Double counting

To account for double counting, where a primary study was included in more than one review, a mapping of primary studies was completed. This produced a corrected cover area (CCA) percentage [19]. Where a primary study overlapped, data from the higher quality review were retained. If both reviews were of the same quality, the data were retained from the newest review. If overlapping data was included in two high quality meta-analysis, the overlapping data was not removed. Once this process was completed, primary studies were re-mapped and a CCA re-calculated.

### Reporting

The results of the four objectives are reported sequentially as objective one to four. Objective two, which compares interventions that have decreased resource use compared to those with no effect is reported as objectives 2a–2d. This is to facilitate easy comparison between interventions that decreased resource use (2a), those that had a mixed effect (2b), those that increased resource use but as the primary aim of the intervention (2c) and those that had no effect (2d).

## Results

**Fig. 1:**
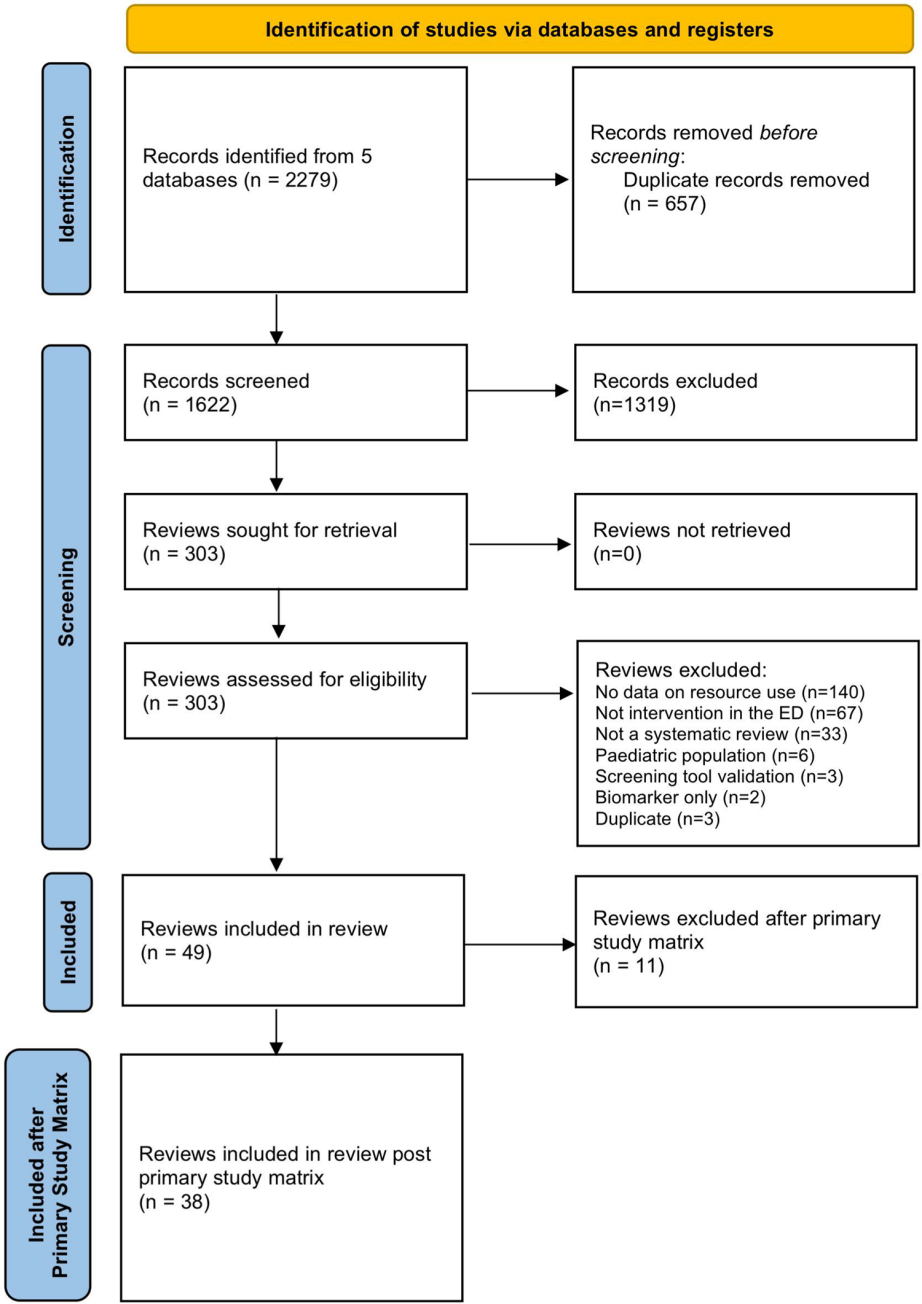
PRISMA flow diagram

**Fig. 2:**
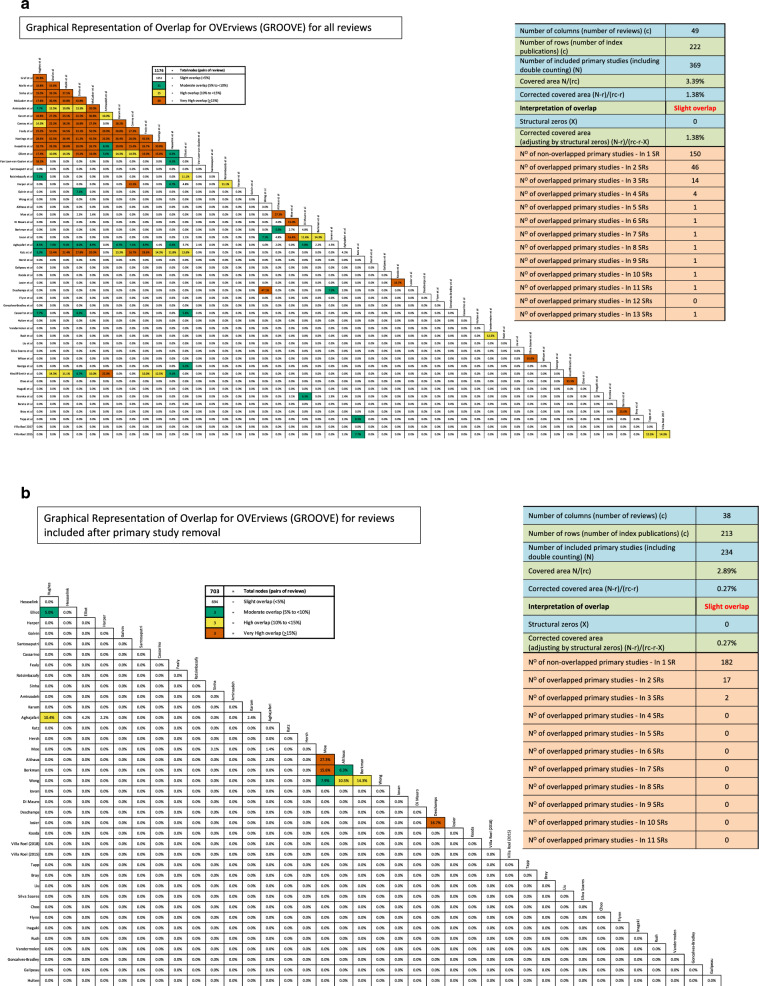
a/b—Primary study matrix, pre and post primary study overlap

A total of 49 eligible reviews were identified from the search, conducted on 16/02/2021 (re-run on 26/01/2022) (Fig. 1). The 49 reviews included data from 369 primary studies. 72 primary studies overlapped. The CCA was 1.38%, demonstrating ‘slight overlap’ overall [19] (Fig. 2a). After removal of overlapping primary studies, not used in meta-analysis data, 11 reviews were removed as primary studies were reported in higher quality reviews. Of the 38 reviews remaining, 213 studies were included, 19 overlapped studies remained. The final CCA was 0.27% (Fig. 2b).

The interrater reliability between the two data extraction reviewers for the first third of reviews was κ = 0.78. This demonstrates ‘substantial’ agreements between reviewers. [15]

Description of included reviews

A detailed description of the 38 reviews is available in Table 1.

**Table 1:** Detailed description of reviews included in overview

Authors (Yr of publication)	Aim of systematic review	Primary population	Intervention	Type of analysis	Primary studies:In review (n)/Reporting resource use (n)/After matrix (n)/% of primary studies included	AMSTAR 2
*Older Adults (n* = *12)*						
Hughes et al. (2019)	To understand how effective emergency department (ED) interventions are in improving clinical, patient experience, and utilization outcomes in older adults (age > 65)	Older adults	Any of discharge planning, case management, medication safety or management geriatric EDs	Meta-analysis	17/12/12/71	H
Hesselink et al. (2019)	Effectiveness of interventions to alleviate emergency department crowding by older adults	Older adults	Any intervention aimed at reducing crowding	Meta-analysis	16/13/4/25	H
Elliott et al. (2022)	Interventions for the discharge of older people to their home from the emergency department	Older adults	Any intervention	Narrative	25/15/9/36	H
Harper et al. (2021)	To understand if providing a fall prevention service in the emergency department is effective	Older patient (with a fall)	Any intervention	Meta-analysis	20/6/6/30	H
Galvin et al. (2017)	Summarise the totality of evidence regarding the predictive value in identifying older adults at risk of adverse outcomes after ED discharge/hospitalisation	Older adults (screening)	Screening tools	Meta-analysis and impact analysis	32/1/1/3	M
Santosaputri et al. (2019)	Efficacy of interventions delivered by staff with geriatric medicine expertise that involve direct patient care (rather than organisational improvement programs), in reducing the hospitalisation of nursing home residents	Nursing home residents	Any intervention by a Geriatric team member	Meta-analysis	16/3/3/19	M
Cassarino et al. (2019)	To explore the impact of early assessment or intervention conducted by interdisciplinary teams with two or more HSCP members in the ED on the quality, safety, and cost-effectiveness of care of adults presenting to the ED	Adults assessed by a non-medical clinician	Interventions conducted in the ED by interdisciplinary teams comprising one or more HSCP members	Narrative	6/3/2/33	M
Fealy et al. (2009)	Analyse data from published studies reporting nursing interventions targeted at older ED attendees, and to provide a critical appraisal of the evidence concerning their effectiveness	Older adults	Gerontologically informed nursing intervention	Narrative	22/8/1/5	L
Ratsimbazafy et al. (2020)	Provide an inventory of all interventions or processes designed to prevent unplanned readmissions or ED visits of older patients presenting to hospital with a fall	Older patient (with a fall)	Any intervention aimed at preventing unplanned admissions	Narrative	6/4/2/33	L
Sinha et al. (2011)	Review of ED-based case management models designed to improve the health, social, and health service utilization outcomes for non-institutionalized older patients within the context of an index ED visit	Older adults	Any intervention	Narrative	20/13/2/10	CL
Karam et al. (2015)	Review the literature on ED-based interventions and examine the evidence on reductions in ED re-visits, hospitalizations, nursing home admissions and deaths among older adults	Older adults	Any intervention aimed at preventing adverse events	Narrative	9/8/2/22	CL
Aminzadeh and Dalziel (2002)	Establish the patterns of use of emergency services among older adults, the risk factors associated with adverse health outcomes in older ED patients, and the effectiveness of intervention strategies targeting this population	Older adults	Any intervention	Narrative	11/3/1/9	CL
*Frequent Attenders (FA) (n* = *7)*						
Moe et al. (2017)	Evaluating the effectiveness of interventions targeting adult frequent ED users at reducing ED visit frequency and improving hospital admissions, mortality, costs, and social outcome	FA	Any intervention	Narrative	31/31/31/100	H
Althaus et al. 2011)	Review the type and effectiveness of interventions to reduce the number of ED visits by frequent users	FA	Case management, less comprehensive case management, Previous notes available to clinician	Narrative	11/11/11/100	H
Berkman et al. ((2021)	Management of High-Need, High-Cost Patients: A Best Fit Framework Synthesis, Realist Review, and Systematic Review	FA	Any intervention	Narrative	40/7/6/15	H
Wong et al. (2020)	Synthesize all available evidence on ED-based interventions aimed at improving the management of recurrent ED utilizing patients with Chronic Non-cancer pain	FA (non-cancer pain)	Any intervention	Narrative	13/12/10/77	M
Deschamps et al. (2021)	Association between supportive interventions and healthcare utilization and outcomes in patients on long-term prescribed opioid therapy presenting to acute healthcare settings	FA (opioids)	Any harm reduction intervention	Meta-analysis	21/13/5/24	L
Iovan et al. (2020)	Interventions aimed at reducing prehospital and emergency care use among superutilizer populations in the United States	FA	Any intervention	Narrative	43/33/16/37	CL
Mauro et al. (2019)	Examine if and how the Case Management (CM) programs are implemented to reduce the number of FU visits to the ED	FA	Case Management	Narrative	14/14/4/29	CL
*Adults in the ED (n* = *3)*						
Aghajafari et al. (2020)	Review care transition interventions (CTIs) for adult patients to understand how effective ED-based CTIs are in reducing return visits to the ED and increasing follow-up visits with primary care physicians	Adult ED patients	Care Transitions	Meta-analysis	42/42/41/98	H
Katz et al. (2012)	Synthesis the effectiveness of ED-based interventions for care coordination with outpatient providers, with the goal of identifying which interventions are effective in improving quality by reducing return visits to the ED and increasing follow-up visits with primary care providers	Adult ED patients	Care co-ordination	Narrative	23/12/4/17	CL
Hersh et al. (2001)	Evaluate the efficacy of telemedicine interventions for health outcomes in two classes of application: home-based and office/hospital based	Adult ED patients	Telemedicine	Narrative	25/1/1/4	CL
*Asthma (n* = *3)*						
Villa-Roel et al. (2018)	In adults presenting to EDs with asthma exacerbations do ED-directed educational interventions involving the provision of individualized Asthma Action Plans, when compared to usual care, reduce the proportion of asthma relapses after an asthma exacerbation?	Asthma	Any educational intervention	Meta-analysis	3/3/2/67	M
Villa-Roel et al. (2016)	Assess and describe the evidence from randomized controlled trials (RCTs) on the effectiveness of ED-directed educational interventions to improve office follow-up visits with PCPs in adults who were discharged from the ED after being treated for acute asthma	Asthma	Any educational intervention	Meta-analysis	5/5/5/100	M
Tapp et al. (2007)	Education interventions for adults who attend the emergency room for acute asthma	Asthma	Any educational intervention	Meta-analysis	13/4/3/23	L
*ED patients on antibiotics (n* = *2)*						
Kooda et al. (2022)	Impact of Pharmacist-Led Antimicrobial Stewardship on Appropriate Antibiotic Prescribing in the Emergency Department	ED patient on antibiotics	Pharmacist	Meta-analysis	24/10/10/42	H
Losier et al. (2017)	To characterize antimicrobial stewardship (AMS) interventions in the ED and to identify stewardship initiatives that result in decreased consequences of antimicrobial use (e.g., Clostridium difficile infection, antimicrobial resistance) and improvement of patient outcomes	ED patient on antibiotics	Antimicrobial Stewardship Intervention	Narrative	43/4/2/67	L
*Atrial Fibrillation (n* = *2)*						
Vandermolen et al. (2018)	Management strategies and decision aids for triaging ED patients with AF, specifically with a plan for selecting patients appropriate for outpatient management	Atrial Fibrillation	Any intervention	Narrative	34/2/1/3	CL
Rush et al. (2020)	Synthesise the evidence examining the impact of transitional care interventions on patient, provider, and health care utilisation outcomes	Atrial Fibrillation	Care Transitions	Narrative	14/7/7/50	CL
*Lower Back Pain (n* = *1)*						
Liu et al. (2018)	Examine the effectiveness and fidelity of interventions aimed at reducing image ordering in the ED for patients with Lower back pain	Lower back pain	Clinical decision support	Meta-analysis	5/2/2/40	M
*Alcohol (n* = *1)*						
Bray et al. (2011a)	To examine effect of screening and brief intervention on outpatient, emergency department, and inpatient health care utilization outcomes	Alcohol	Screening tools	Meta-analysis	29/4/4/14	CL
*Palliative Care (n* = *1)*						
da Silva Soares et al. (2016)	Effectiveness of ED-based Palliative Care interventions on hospital admissions, length of stay, symptoms, quality of life, use of other health care services, and Palliative Care referrals for adults with advanced disease	Palliative Care	Any intervention by a palliative care team member	Narrative	5/2/2/40	L
*Risky Behaviour (Domestic Violence) (n* = *1)*						
Choo et al. (2012)	Evaluate the evidence for use of computer technologies to assess and reduce high-risk health behaviours in emergency department (ED) patients	Risky Behaviour	Computer technology	Narrative	20/2/2/10	L
*Shared decision-making (n* = *1)*						
Flynn et al. (2012b)	Evaluate the approaches, methods, and tools used to engage patients or their surrogates in shared decision-making in the ED	Shared decision-making	Shared decision-making	Narrative	5/2/2/40	H
*Mental Health (n* = *1)*						
Inagaki et al. (2019)	Effect of ED-initiated active contact and follow-up interventions on the risk of a repeat suicide attempt within 6 months in patients admitted to an ED for suicidal injury	Mental Health (Suicide)	Any intervention	Meta-analysis	34/7/7/21	L
*Primary Care ED patients (n* = *1)*						
Goncalves-Bradley et al. (2018)	To assess the effects of locating primary care professionals in hospital EDs to provide care for patients with non-urgent health problems, compared with care provided by regularly scheduled EPs	Primary Care patients in ED	GP review in ED	Narrative	4/2/2/50	H
*ED Short Stay Unit (n* = *1)*						
Galipeau et al. (2015)	Evaluate the effectiveness and safety of ED short-stay units compared with care not involving short-stay units	Adults in ED short stay unit	Short stay units	Meta-analysis and Narrative	5/5/5/100	H
*Chest Pain (n* = *1)*						
Hulten Edward et al. (2013)	Evaluate RCTs of ED triage of acute chest pain and compare CCTA and usual care for the incidence of coronary angiography, coronary revascularization, death, nonfatal myocardial infarction, repeat ED evaluations for chest pain, re-admission to the hospital for ACS, LOS, and cost	Chest pain	CCTA—coronary computed tomography angiography (CCTA)	Meta-analysis	4/4/4/100	L

*H* high, *M* moderate, *L* low, *CL* critically low

Methodological quality of included reviews

**Fig. 3:**
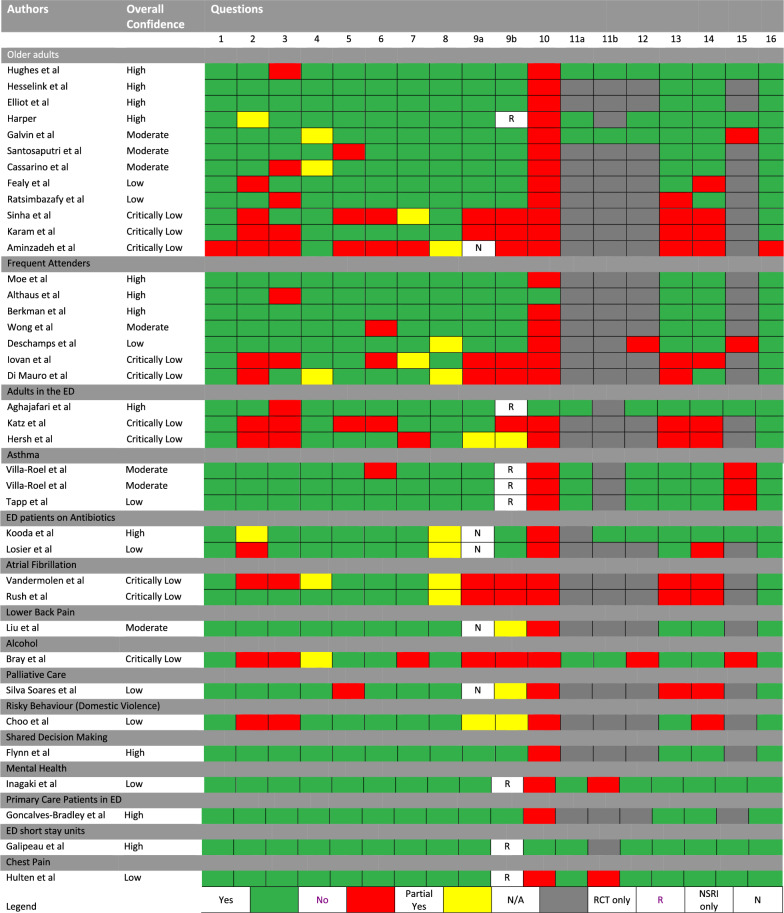
AMSTAR-2 assessment

The itemised results of the AMSTAR-2 assessment are outlined in Fig. 3. The overall confidence in the included reviews was defined as high in 12, moderate in seven, low in nine and critically low in 10 reviews (n = 38).

Risk of bias of primary studies included in reviews

The overall impact of the risk of bias of primary studies in each review is covered by items nine, 12, 13 and 15 in the AMSTAR-2 assessment (Fig. 3).

When analysed individually 72.7% (n = 24/31) of reviews used a satisfactory technique for assessing RoB in individual RCTs, and 56.7% (n = 17/30) for non-randomised studies of interventions (NRSI) (item nine, online supplement-2). In the 13 studies that performed a meta-analysis, 84.6% (n = 11/13) assessed the impact of RoB of individual studies on the meta-analysis (item 12 online supplement-2). Most reviews (71.1%, n = 27/38) accounted for RoB during the interpretation of the results, but only 53.1% (n = 7/13) of reviews investigated publication bias when indicated (items 13 and 15, online supplement-2).

Where available, the individual RoB assessment for the primary studies in each review is available in the online supplement-3.

### Outcome 1: Reviews which evaluate ED interventions and report subsequent healthcare resource use as an outcome

In the 38 reviews, 37 unique interventions were analysed. Table 2 outlines the direction of effect of interventions, grouped by host population (n = 15) and specific resource use measured (n = 9). This resulted in 30 different intervention-population-resource use combinations. ED based interventions decreased subsequent healthcare resource use in 23.3% (n = 7/30) of the intervention-population-resource use combinations, had a mixed effect in 10% (n = 3/30), increased scheduled follow-up (aim of the interventions) in 20% (n = 6/30) and had no effect in 40% (n = 12/30). For 6.6% (n = 2/30) it was not possible to report an effect.

**Table 2:** Effect of interventions by population-intervention-resource use combination

Population– Resource use	Interventions	Explanation
*Decreased subsequent healthcare resource use*		
Frequent Attenders – ED revisit	Care plans Case management Social work home visits Diversion strategies to nonurgent care Printout case notes Medical Care Plan** Care Co-ordination** Disease Management**	In ED frequent attendance patients, interventions, decreased ED revisits. This is based on high confidence data from 3 reviews (Moe et al*.* 2017) Median rate ratio was 0.63 (IQR = 0.41 to 0.71), general effect of interventions was to decrease ED visits post-intervention. Data from 10/31 primary studies AMSTAR II of review = High. GRADE of outcome = not reported. RoB = 7 Moderate, 3 High (Berkman et al*.* 2021) – Reduction in ED revisit 3/4 RCT = reduction, 1/4 RCT = no difference, 1/2 OBS = reduction. 1/2 OBS samples = reduction one control group and no difference with one control group AMSTAR II of review = High. GRADE of outcome = Moderate RoB of primary studies = Low = 1, Some Concerns = 3, High = 2 (Althaus et al*.* 2011)– 7/11 decrease primary studies, 1 increase primary study, 2 not reported AMSTAR II of review = High. GRADE of outcome = not reported. RoB = reported individually see online supplement This data is supported by 1 Moderate confidence reviews (Wong et al*.* 2020). Reduction in visits between 48.4 and 89.5% GRADE = not reported. RoB/Quality, 2 = moderate quality 3 = low quality primary studies Supported by 3 Critically Low confidence reviews ((Iovan et al*.* 2020), (Mauro et al*.* 2019), (Deschamps et al*.* 2021)) 11/17 decrease primary studies, 7/17 No effect (1 study reported twice), 2/4 decrease primary studies, 1/4 No effect, 1/4 Unable to comment 2/5 decrease primary studies, 3/5 Unable to comment
Shared decision-making – ED Revisit	Provision of pre-test probability	In patients presenting with chest pain, interventions, decreased 7-day ED revisit rate. This is based on high confidence data from 1 review (Flynn et al*.* 2012b), 1/1 decrease primary study (RCT) AMSTAR II of review = High. GRADE of outcome = NR. RoB = Low, Quality of primary study = High
Alcohol – ED revisits	Screening and brief interventions	In patients screened for alcohol, screening and brief interventions, decreased ED revisits. This is based on critically low confidence data from 1 review (Bray, Cowell and Hinde, 2011b), 3/4 decrease primary studies. 1/4 unable to comment AMSTAR II of review = Critically low. GRADE of outcome = NR. Qualitative Methodological Scores = 13, 13, 14 (high)
Frequent Attenders – Inpatient admissions	ED- initiated patient navigation program Emergency Room Decision- Support Medical Care Plan** Care Co-ordination** Disease Management**	In ED frequent attendance patients, interventions, decreased inpatient admissions. This is based on high confidence data from 1 review (Berkman et al*.* 2021)—Effect = Decrease, based on 1 × RCT, 1 × OBS study AMSTAR II of review = High. GRADE of outcome = Low. RoB = RCT – Low, OBS – Some Concerns This data is supported by Critically Low confidence data from (Iovan et al*.* 2020)—9 no effect, 9 decrease
Older Adults who fell - Hospital Admission	Interdisciplinary team	(Harper et al*.* 2021), RR 0.76; 95% CI 0.64–0.90, AMSTAR II of review = High. GRADE of outcome = Moderate. RoB = Moderate to strong quality (RoB assessment included)
Short Stay Units in the ED – Hospital Admissions	ED short stay units	For patients in the ED, ED short stay units had a decreased Hospital admission. This is based on high confidence data from 1 review (Galipeau et al*.* 2015)—3/3 primary studies positive AMSTAR II of review = High. GRADE of outcome = low. RoB = Moderate
Shared decision-making – Testing	Chest pain decision aid Provision of pre-test probability	In patients presenting with chest pain, interventions, decreased testing. This is based on high confidence data from 1 review (Flynn et al*.* 2012b), 1/1 primary study for decreased cardiac testing at 30 days (decision aid), 1/1 positive primary study for decreased thoracic imagine (pre-test probability) AMSTAR II of review = High. GRADE of outcome = NR. RoB = Low × 2, Quality of primary study = High × 2
Frequent Attenders – Cost	Care plans Case management Social work home visits Diversion strategies to nonurgent care Care coordination and community health worker program Emergency Room Decision- Support (ERDS) program Pain protocol Individual Care Plan**	In ED frequent attendance patients, interventions, have a decreased Healthcare Costs. This is based on high confidence data from 2 reviews (Moe et al*.* 2017)—11 decrease (RoB = 4 Moderate, 7 High)., 1 increase (outpatient costs, RoB = moderate), 1 no effect (non-ED costs, RoB = High) AMSTAR II of review = High. GRADE of outcome = NR (Berkman et al*.* 2021)—Effect = decrease, based on 2 of 3 RCT samples had favourable findings (RoB = 2 × Some Concerns), 1 of 3 RCT samples found no difference (RoB = 1 × Some Concerns), 1 of 1 OBS sample found no difference (RoB = 1 × Some Concerns) AMSTAR II of review = High. GRADE of outcome = Low This data is supported by 1 Moderate confidence review (Wong et al*.* 2020) which reported a reduction in costs in 3 studies GRADE = not reported. RoB/Quality. 1 = moderate quality 2 = low quality 1 Critically Low confidence reviews (Mauro et al*.* 2019) reported a reduction in cost in 1 primary study (CASP Quality score 11/11)
*Mixed effect on resource use*		
Lower Back Pain – ED revisit	MDT protocols Clinical decision support	In patients with lower back pain, interventions, had a decrease to no effect on ED revisits. This is based on moderation confidence data from 1 review (Liu et al*.* 2018)– 2 before and after primary studies. MDT protocols aimed at decreasing imaging for lower back pain decreased ED revisits, whilst clinical decision support had no effect AMSTAR II of review = Moderate. GRADE of outcome = NR. Before and After Quality Assessment = low × 2
Older Adults who Fell - Hospital Admission	Interdisciplinary team	A non-significant reduction (P = 0.07) with intervention (RR 0.85; 95% CI 0.72–1.01, I2 0%). Heterogeneity: Tau(2) = 0.00, CHI(2) = 2.13, df = 4, test for overall effect 1.92 (p = 0.06)
Mental Health (acute suicidal ideation) – Psychiatric Admissions	Active follow-up and contact interventions	In patients presenting with acute suicidal ideation, interventions, both decreased and had no effect on Psychiatric Admissions. This is based on low confidence data from 1 review (Inagaki et al*.* 2019), For psychiatric admissions at 12 months, 1 study had a decrease effect (RoB L = 3 U = 2 H = 2). 2 studies had no effect (RoB L = 4 U = 3 H = 0, L = 4 U = 1 H = 2) AMSTAR II of review = Low. GRADE of outcome = NR. RoB = see above
Short Stay Units – ED revisit/Hospital Readmission	ED short stay units	For patients in the ED, ED short stay units had a decrease to no effect on ED revisit/Hospital readmission. This is based on high confidence data from 1 review (Galipeau et al*.* 2015)—2/4 decrease primary studies, 2/4 no effect primary studies AMSTAR II of review = High. GRADE of outcome = low. RoB = Moderate
*Increase in scheduled follow-up*		
Frequent Attenders – Primary Care Attendance	Patient navigation for ED patients Emergency Room Decision- Support (ERDS) program	In ED frequent attendance patients, interventions, increased Primary Care visits. This is based on high confidence data from 1 review (Berkman et al*.* 2021) – Effect = Increase, based on 1xRCT, 1 × OBS study AMSTAR II of review = High. GRADE of outcome = Low. RoB = RCT – Low, OBS – Some Concerns
Asthma – Primary care follow-up	*Educational interventions*: Arranged follow-up Follow-up phone calls Faxed letters Oral steroids Asthma action plans	In asthma patients, educational interventions increased scheduled follow-up rates with Primary Care Practitioners (aim of interventions). This is based on moderate confidence data from 1 study (Villa—Roel et al*.* 2016) Risk Ratio = 1.6; 95% CI 1.31 to 1.87 AMSTAR II of review = Moderate. GRADE of outcome = NR. RoB = Unclear
Adults in the ED – Follow-up with specialist or primary care providers	*Care Transition Interventions defined as*: Educational support (face-to-face, video-based or telephonic) Reminders (mailed, text or telephonic) Appointment scheduling ED-based discharge instructions Case management programs	In ED adult patients, care transition interventions, improve the rate of follow-up with specialist or primary care providers. This is based on high confidence data from 1 review (Aghajafari et al*.* 2020) 20 studies (8178 patients). ED-based CTIs increased odds of follow-up versus usual care (OR 1.79, 95% CI 1.43,2.24) AMSTAR II of review = High. GRADE of outcome = Low. RoB of primary studies = Low in 11/40 This data is supported by 1 critically low confidence review (Katz et al*.* 2012) Based on 5/5 studies that demonstrated increased follow up (3/5 positive, 1/5 no effect, 1/5 NR). Grade and RoB not reported. JADAD score 3/5 and 2/5 in the two Randomised studies
Frequent Attenders – Outpatient visits	Care plans Case management Social work home visits Diversion strategies to nonurgent care Printout case notes	In ED frequent attendance patients, interventions, have increased outpatient visits. This is based on high confidence data from 2 reviews (Althaus F. et al*.* 2010)—Effect = 2 studies increased outpatient visits, as per aim AMSTAR II of review = High. GRADE of outcome = not reported. Quality Criteria for NCBA studies = "Y = 6 U = 2 N = 2, Y = 7 U = 1 N = 2" (Moe et al*.* 2017)—Effect = 6 studies increased outpatient attendances (RoB = 4 × Moderate, 2 × High), 2 no effect (RoB = 2 × High), 1 decreased outpatient attendances (RoB = 1 × Moderate). The aim of interventions was not reported AMSTAR II of review = High. GRADE of outcome = not reported *(Unable to establish if this was the aim of interventions in Moe et al.)
Lower Back Pain – Physio & Rehab	MDT protocols	In patients with lower back pain, interventions, increased the use of physiotherapy and rehabilitation services visits This is based on moderate confidence data from 1 review (Liu et al*.* 2018) – 1 before and after primary studies. MDT protocols aimed at decreasing imaging for lower back pain increased use of services AMSTAR II of review = Moderate. GRADE of outcome = NR. Before and After Quality Assessment = low × 1
Risky Behaviour (Domestic Violence) – Increased Referral	Patient and physician notification	In patients presenting with a domestic violence related issue, interventions, increased referrals to services. This is based on low confidence data from 1 review (Choo et al*.* 2012), 1/2 increase primary studies. 1/2 unable to comment AMSTAR II of review = low. GRADE of outcome = NR. Quality of primary study = moderate
*No effect on subsequent healthcare resource use*		
Older Adults – ED revisits	Case management Discharge planning Complex geriatric assessment	In Older Adult ED patients, interventions had no effects on ED revisits. This is based on high confidence data from 4 reviews (Hughes et al*.* 2019), RR = 1.13; 95% CI 0.94—1.36 AMSTAR II of review = High. GRADE of outcome = high. RoB of primary studies = Low to High (Harper et al*.* 2021), RR 0.85; 95% CI 0.72–1.01 AMSTAR II of review = High. GRADE of outcome = Low. Quality of primary studies = Moderate to Strong (RoB assessment included) (Hesselink, Sir and Schoon, 2019), 1/4 Primary study positive effect at 1 and 3 months, 4/4 Primary studies = No effect, AMSTAR II of review = High. GRADE of outcome = NR. RoB of primary studies = Moderate to high (Elliott et al*.* 2022), 5/8 primary studies = Positive effect, 3/8 = No effect AMSTAR II of review = High. GRADE of outcome = NR. RoB = Low to Moderate (only in randomised trials) This data is supported by 3 Moderate confidence reviews ((Galvin et al*.* 2017), (Santosaputri E., Laver K., and To T., 2019), Cassarino), 2 Low confidence reviews ((Fealy et al*.* 2009), (Ratsimbazafy et al*.* 2020)) and 3 Critically Low confidence reviews ((Sinha et al*.* 2011), (Aminzadeh and Dalziel, 2002), (Karam et al*.* 2015)) 5/10 decrease primary studies, 4/10 No effect primary studies, 1/10, Unable to comment primary studies
Adults in the ED – ED revisits	*Care Transition Interventions defined as*: Educational support (face-to-face, video-based or telephonic) Reminders (mailed, text or telephonic) Appointment scheduling ED-based discharge instructions Case management programs	In ED adult patients, care transition interventions, have no effect on ED revisits. This is based on high confidence data from 1 review (Aghajafari et al*.* 2020) 20 studies (8048 patients). ED-based CTIs had no effect on ED revisit (OR 1.01, 95% CI 0.86, 1.20), (experimental group events = n = 845, control group events = n = 832) AMSTAR II of review = High. GRADE of outcome = Low. RoB of primary studies = Low in 12/20 This data is supported by 1 Critically low confidence review (Katz et al*.* 2012). Based on 3/5 (1xRCT, 2xOBS) studies that demonstrated no effect on ED revisits. Grade and RoB not reported. JADAD score 3/5 RCT In adult patients, telemedicine interventions had no effect on ED revisit based on Critically low narrative data from (Hersh et al*.* 2001), based on 1 RCT. Grade and RoB not reported
Asthma – ED revisit	*Educational interventions*: Arranged follow-up Follow-up phone calls Faxed letters ral steroids and transport vouchers Asthma action plans	In asthma patients, educational interventions, had no effect on Asthma relapses (including ED revisits). This is based on moderate confidence data from 1 study (Villa—Roel et al*.* 2016)) – Risk Ratio = 1.3 (95% CI 0.82 to 1.98) AMSTAR II of review = Moderate. GRADE of outcome = NR. RoB = Unclear This data is supported by 1 moderate confidence narrative review (Villa-Roel et al*.* 2018) based on one high RoB and one low RoB studies (one had a decrease effect, the other an increase effect for AAP and % relapses) This evidence is supported 1 low confidence review. (Tapp, Lasserson and Rowe, 2007) No effect on ED revisit. Grade of outcome = low. Based on three RCTs with mixed RoB
Antibiotics – ED revisit	Pharmacist lead algorithm Pharmacist culture follow-up Pharmacist presence	In ED patients, pharmacist interventions, had no effects on ED revisits. This is based on high confidence meta-analysis data from 1 review (Kooda, Canterbury and Bellolio, 2022) OR of 0.65 (95% CI 0.39 to 1.10) (Tau2 = 0.42, CHI2 = 53.57, df = 9 P < 0.00001, I2 = 83%, Z = 1.59 p = 0.11) AMSTAR II of review = High. GRADE of outcome = NR. Newcastle–Ottawa RoB Moderate 9/10, High 1/10, NIH Quality Score Fair = 7/10, Good 3/10 This data is supported by 1 Low confidence review (Losier et al*.* 2017). 1 study (high RoB) demonstrated a decrease effect, 1 study (high RoB) demonstrated a positive effect on ED revisit
General Practitioners in the ED – ED revisit	GPs in the ED	For patients in the ED, being seen by a GP had no effect on ED Revisits. This is based on high confidence data from 1 review (Gonçalves-Bradley et al*.* 2018)—1 primary study. 17% (95% CI 15.7% to 18.8%) of patients seen by a GP, and 18% (95% CI 16.3% to 19.5%) of patients seen by an Emergency Physician re-attending the ED for the same problem within 30 days of index visit AMSTAR II of review = High. GRADE of outcome = very low. RoB = L = 3 U = 8 H = 3
Adults with chest pain – ED revisit	CCTA	For chest pain, CCTA had no effect on ED revisit. This is based on low confidence data from 1 review. (Hulten Edward et al*.* 2013) Pooled weighted odds ratio (range) 0.94 (0.67–1.31, p 0.70) I2 = 0.0%, p = 0.68 AMSTAR II of review = Low. GRADE of outcome = NR. RoB = Low-Unclear
Mental Health (acute suicidal ideation) – ED Contacts	Active follow-up and contact interventions	In patients presenting with acute suicidal ideation, interventions, had a no effect on ED contacts. This is based on low confidence data from 1 review (Inagaki et al*.* 2019), 1 primary study showed no effect AMSTAR II of review = Low. GRADE of outcome = NR. RoB = L = 4 U = 1 H = 2
Mental Health (acute suicidal ideation) – GP Contacts	Active follow-up and contact interventions	In patients presenting with acute suicidal ideation, interventions, had a no effect on GP contacts. This is based on low confidence data from 1 review (Inagaki et al*.* 2019), active contact resulted in a reduction at 3 months but this was reversed to an increase at 12 months (n = 1, RoB L = 4 U = 3 H = 0). 2 other studies (presented in 3 papers) showed no effect (n = 3 RoB L = 5 U = 1 H = 1, L = 5 U = 1 H = 1, L = 4 U = 2 H = 1). AMSTAR II of review = Low. GRADE of outcome = NR. RoB = see above
General Practitioners in the ED – GP visits	GPs in the ED	For patients in the ED, being seen by a GP had no effect on GP visits. This is based on high confidence data from 1 review (Goncalves-Bradley D. et al*.* 2018)—2 primary studies. No effect AMSTAR II of review = High. GRADE of outcome = very low. RoB = L = 3 U = 8 H = 3, L = 5 U = 4 H = 5
Mental Health (acute suicidal ideation) – Psychiatric Contacts	Active follow-up and contact interventions	In patients presenting with acute suicidal ideation, interventions, both decreased and increased on Psychiatric contacts. This is based on low confidence data from 1 review (Inagaki et al*.* 2019), For psychiatric contacts at 12 months, 1 study had a decrease effect (L = 1 U = 1 H = 4), 1 had an increase effect (RoB L = 5 U = 1 H = 1) AMSTAR II of review = Low. GRADE of outcome = NR. RoB = see above
Older Adults – Hospital re-admissions	Case management Discharge planning Complex geriatric assessment	In Older Adult ED patients, interventions had no effect on Hospital re-admissions. This is based on high confidence data from 2 reviews (Hughes et al*.* 2019), Relative risk [RR] = 0.96; 95% CI 0.51–1.83 AMSTAR II of review = High. GRADE of outcome = Low. RoB of primary studies = Low to High (Elliott et al*.* 2022), 2/2 primary studies = No effect AMSTAR II of review = High. GRADE of outcome = NR. RoB = Low 1/2, NR in 1/2 This data is supported by 2 Moderate confidence reviews ((Cassarino et al*.* 2019; Santosaputri E., Laver K., and To T., 2019)) and 2 Low confidence reviews ((Ratsimbazafy et al*.* 2020; Fealy et al*.* 2009)) 4/7 decrease primary studies, 3/7 No effect primary studies
Adults with chest pain – Hospital Admission	CCTA	For chest pain, CCTA had no effect on hospital admissions. This is based on low confidence data from 1 review. (Hulten Edward et al*.* 2013) Pooled weighted odds ratio (range) 1.20 (0.67–2.16, p 0.50) I2 = 0.0%, p = 0.68) AMSTAR II of review = Low. GRADE of outcome = NR. RoB = Low- Unclear
Adults in the ED – Hospital Re-admission	*Care Transition Interventions defined as*: Educational support (face-to-face, video-based or telephonic) Reminders (mailed, text or telephonic) Appointment scheduling ED-based discharge instructions -Case management programs	In ED adult patients, care transition interventions, had no effect on hospital re-admissions. This is based on high confidence data from 1 review. (Aghajafari et al*.* 2020) 13 studies (5742 patients). ED-based CTIs had no effect on hospital admissions (OR 0.99, 95% CI 0.86,1.14) AMSTAR II of review = High. GRADE of outcome = Low. RoB of primary studies = Low in 11/40 This data is supported by a 1 Critically low confidence review ((Katz et al*.* 2012)). Based on 1/5 (1xOBS) studies that demonstrated increased hospitalisations. Grade and RoB not reported
*Unclear aim of intervention/not possible to evaluate*		
Alcohol – Outpatient Resource Use	Screening and brief interventions	In patients screened for alcohol, screening and brief interventions, increased outpatient resource use. This is based on critically low confidence data from 1 review (Bray, Cowell and Hinde, 2011b), 2/4 increased resource use. 2/4 unable to comment AMSTAR II of review = Critically low. GRADE of outcome = NR. Qualitative Methodological Scores = 13, 12 (high) *(Unable to establish if this was the aim of interventions)
Palliative Care – ED revisit	N/A	From 1 review it is not possible to comment on the effect of Palliative Care ED interventions on subsequent healthcare resource use. (da Silva Soares, Nunes and Gomes, 2016)

^**^Data from critically low confidence review

The 15 populations, dictated by cohorts reported in reviews, were older adults (n = 12), frequent attenders (n = 7), ED adults (n = 3), asthma (n = 3), atrial Fibrillation (n = 2), patients on antibiotics (n = 2), alcohol related (n = 1), lower back pain (n = 1), risky behaviour (n = 1), shared decision making (n = 1), mental health (n = 1), primary care patients in ED (n = 1), ED short stay unit patients (n = 1), chest pain (n = 1) and palliative care (n = 1).

### Outcome 2a: Interventions that decreased subsequent healthcare resource

Only data from high or moderate confidence reviews are reported below for all outcomes below. Table 2 includes additional data from low or critically low confidence reviews for reference.

#### ED revisits

##### Frequent attenders

Three high confidence reviews [20–22] demonstrated a decrease in ED revisits when care plans, case management, social work home visits, diversion strategies to non-urgent care, printout case notes were used in the patients defined as frequent attenders. A moderate GRADE was reported by Berkman et al. [21], indicating certainty that the true effect of the interventions were a reduction in ED revisits. No GRADE was reported by the other two reviews.

This data is supported by moderate confidence data from Wong et al. [23]. Data from five studies (two moderate and three low quality) demonstrated a reduction in ED revisits between 48.4% and 89.5%. Interventions were care co-ordination, pain protocols, pain contract (present twice) and behavioural interventions.

##### Patients presenting with chest pain

Data from a high confidence review by Flynn et al. [24] demonstrated that the provision of pre-test probability to patients and clinicians decreased 7-day ED revisit rate. Based on evidence from one study with low RoB. [25]

#### Hospital admissions

##### Frequent attenders

Based on a high confidence review by Berkman et al. [21], which reported one low RoB RCT [26] (n = 100) and one observational study [27] (n = 14 140) with “some” RoB concerns, ED-initiated patient navigation programme and decision-support were found to decrease hospital admissions in frequent attenders.

#### Hospital re-admissions

##### Older adults

Based on one high confidence review by Harper et al. [28], reporting data from two strong and four moderate quality RCTs (n = 2493), Interdisciplinary team interventions reduced hospital re-admission in older adults who fell, with a relative risk (RR) of hospital re-admission of 0.76 (95% CI 0.64–0.90). The GRADE was reported as moderate.

#### Testing and cost

Testing and cost were identified as additional healthcare resource use outcomes. These were not defined a-priori and are therefore presented in the online supplement-4.

### Outcome 2b: Interventions that had a mixed effect on subsequent healthcare resource

#### ED revisit

##### Patients with lower back pain

A moderate confidence review from by Liu et al. [29], based on two studies with ‘low’ ‘Before and After Quality Assessment’ (BAQA) score, reported that multi-disciplinary team protocols aimed at decreasing imaging for lower back pain decreased ED revisits, whilst clinical decision support had no effect on ED revisits.

#### Hospital re-admissions

##### Older adults

A high confidence review by Hughes et al. [30] demonstrated that case management, transitions of care, medication management and discharge planning interventions did not have an effect on hospital re-admissions in a general older population. This is based on meta-analysis data from seven RCTs (n = 4806), reporting a RR of hospital re-admission of 0.96 (95% CI 0.51–1.83). The GRADE was low. Another high confidence by Elliot et al. [31], reported that MDT assessment demonstrated no effect in older adults on hospital re-admission. Based on data from two studies (1 × low RoB, 1 × Not Reported). This is in contrast to the review by Harper et al. [28], reported above, which showed interdisciplinary team interventions reduced hospital re-admission in older adults who fell.

Based on high confidence data from Galipeau et al. [32], short stay ED units resulted in decreased to no effect on hospital readmissions and ED revisits in adult ED patients (GRADE = Low, RoB = Moderate).

### Outcome 2c: Interventions that increased scheduled healthcare resource as their aim

Some ED interventions were designed to increase scheduled resource use as per intervention design or national guidance. For example, UK guidelines advise GP follow-up within two days of ED attendance with asthma [33]. Detailed results are available in the online supplement-5. In summary, interventions designed for ED frequent attenders to seek more ‘appropriate’ healthcare options other than the ED, resulted in increased primary care visits as intended [21]. In patients presenting to the ED with asthma, educational interventions increased follow-up rates with a primary care practitioner as intended [34]. Care transition interventions improved the rate of follow-up with primary care or specialist providers in adult ED patients [35]. Case management interventions in ED frequent attenders increased outpatient visits as intended [22]. Finally, multi-disciplinary team protocols aimed at decreasing imaging for lower back pain, increased the use of physiotherapy and rehabilitation services as planned. [29]

### Outcome 2d: Interventions that have no effect on subsequent healthcare resource

These are reported in detail in Table 2. In summary, of the 12 intervention-population-resource use combinations, 6 reported ED revisits, the other six reported ED contacts, GP contacts, GP visits, psychiatric contacts, hospital admission and hospital re-admission.

### Outcome 3: Theoretical concepts that underpin successful interventions

Reviews more frequently reported increased resource use for scheduled follow-up when that was the aim of the intervention, compared to no effect for unscheduled care (i.e., no decrease in unscheduled care) when that was the aim of the intervention. This is based on eight populations (supplement Table 5) that reported scheduled follow-up, of which 87.5% (n = 7/8) reported interventions that increased scheduled follow-up. When compared to 23 unscheduled resource outcomes (from 13 populations), only 30.4% (n = 7/23) reported interventions that decreased unscheduled care.

Further analysis of the seven populations that increased scheduled follow-up, demonstrated six populations that reported both scheduled (e.g., planned GP follow-up) and unscheduled resource use (e.g., ED revisits) as outcomes from the same intervention. Interventions increased scheduled and decreased unscheduled care in two cohorts (frequent attenders and alcohol cohorts); increased scheduled resource use but no effect on unscheduled care in three cohorts (adults in the ED, asthma, alcohol) and increased scheduled resource use but had a mixed effect on unscheduled care in the lower back pain population.

In the 23 unscheduled resource use outcomes reported above, 17.4% (n = 4/23) decreased or had no effect on unscheduled resource use, 47.8% (n = 11/23) had no effect and one could not be analysed.

### Outcome 4: Variability in definitions of downstream healthcare resource

The most common resource use reported was ED Revisit, reported in 36 of 38 reviews (online supplement-4). Overall, there were only nine distinct types of resources identified—ED revisit, hospital admission (including psychiatric), hospital re-admission, GP follow-up, community referral (physiotherapy, rehabilitation community psychiatry), cost, outpatient visits (including psychiatric), general resource use and testing. EMS use or telephone triage (e.g., 111 services in the UK) were not measured in any review.

There were more than 23 different time intervals for follow-up reported across primary studies. The most common was 12 months (n = 52/216), followed by the combined group of 28 days, four weeks, 30 days and one month (n = 44/216) and then six months (n = 40/216) (Table 3). Thirteen primary studies measured follow-up over a period greater than 18 months.

**Table 3:** The duration of follow-up reported by primary studies in reviews

Review grouped by population N^o^ of primary studies in review reporting follow-up (N^0^ of primary studies with > 1 f/u period)	Days										D/W/M			Months										
	1	2	3	5	7	8	14	21	35	45	4W/28 D	30 D/1 M	6W	2	3	4	6	7	9	10	12	18	> 18	
*Older adults*																								
Hughes et al. (2019)	12 (4)							1		1		3	6			2	1	1			1	1	1	
Hesselink et al. (2019)	4 (3)					2							3			1		2						
Elliott et al. (2021)	9 (8)	1					1	1					3			4	1	4		1		4		1
Harper et al. (2021)	6																	2				4		
Galvin et al. (2017)	1												1											
Santosaputri et al. (2019)	4											1	1			1		1				1		
Cassarino et al. (2019)	1																					1		
Fealy et al. (2009)	1											1												
Ratsimbazafy et al. (2020)	2												1			1								
Sinha et al. (2011)	2																1					1		
Karam et al. (2015)	1														1	1								
*Frequent attenders*																								
Althaus F. et al. (2010)	11																	1				9		1
Berkman et al. (2021)	5																	1	1			3		
Deschamps et al. (2021)	5																	1						4
Iovan et al. (2020)	14 (2)															2		4				7	2	1
Mauro et al. (2019)	4																					1	1	2
*Adults*																								
Aghajafari et al. (2020)	41 (7)	1	2	2	2	2		1	1			1	10	1	1	4	1	11		1		5	1	2
Katz et al.( 2012)	4 (1)					1		1				1			1			1						
*Asthma*																								
Villa-Roel et al. (2018)	2 (1)							1										2				1		
Villa-Roel et al. (2016)	5 (3)								1			1	2	1	1	2		1				2		
Tapp, Lasserson and Rowe, (2007)	3 (1)																1	2				1		
*Antibiotics*																								
Losier et al. (2017)	2												2											
*Atrial fibrillation*																								
Vandermolen et al. (2018)	1												1											
Rush et al. (2020)	7 (3nr)												1			2		1						
*Lower back pain*																								
Liu et al. (2018)	2																	1				1		
*Alcohol*																								
Bray et al. (2011a)	4																					4		
*Palliative care*																								
da Silva Soares, Nunes and Gomes, (2016)	2 (1)												1					1				1		
*Domestic Violence*																								
Choo et al. (2012)	2 (1nr)															1								
*Shared decision-making*																								
Flynn et al. (2012b)	2										1		1											
*Mental Health*																								
Inagaki et al. (2019)	7																	1				4		2
*Primary Care ED Patients*																								
Goncalves-Bradley D. et al. (2018)	2					1							1											
*ED Short Stay Unit*																								
Galipeau et al. (2015)	5												1		1	1		1				1		
*Chest pain*																								
Hulten Edward et al. (2013)	4												1					1						
Totals		2	2	2	2	6	1	5	2	1	1	8	36	2	5	22	5	40	1	2	1	52	5	13

*D* days, *W* week, *M* month, *nr* not reported

## Discussion

This overview provides a contemporary map of ED based interventions that impact upon subsequent healthcare resource after ED discharge.

It reports that 40% of interventions have no effect on resource use, however there is evidence within specific population-intervention cohorts (e.g. frequent attenders cohorts or shared decision making interventions) that interventions decrease subsequent healthcare resource use. The data can be practically utilised by intervention developers to review the available evidence of ED based interventions in specific patient cohorts and for specific resource outcomes. It will allow a streamlining of future efforts in those interventions where reliable evidence exists and prevent the repeated trials of interventions which have little evidence of impact.

### Limitations

It is important to consider the results through the lens of overview methodology, which is to provide an overall summary of the available data.

This study was limited by two protocol deviations. Firstly, due to resource limitations data extraction was not completed in duplicate. Duplicate data extraction only occurred for the first third of reviews. At this point an inter-rater reliability was calculated and deemed sufficiently high (κ = 0.78) to continue with single data extraction. Secondly, if risk of bias assessments or GRADE ratings were not reported in the review, they were not calculated as originally specified in the protocol. Again, this was due to resource and time limitations. Both these deviations increase the possibility of bias into the overview. Finally, the search was limited to the English language which increases the chance of language bias.

### Strengths

Despite the limitations, the alignment with overview methods was a key strength of this study. The use of Groove methodology, to account for primary study overlap, was a significant step forward in overview methods that has not, to the authors knowledge, been used previously in emergency care overviews [8, 9, 36, 37]. Our evidence suggests that whilst the overall confidence one can have in review evidence is improving, especially in more recent reviews, there remains consistent heterogeneity in reporting as outlined by Conneely et al*.* [37]

When compared to the results of previous work in this area, three of the four previous overviews of ED based interventions concluded that the evidence base was either “weak” [9, 36] or conclusions were difficult to identify due to the “significant heterogeneity in methods, intervention content and reporting of outcomes” [37].

An understanding of the subsequent healthcare resource use associated with ED based interventions remains important due to the significant pressures across the entire healthcare sector worldwide. Data from this overview highlights the need for a standardised set of outcome measures and follow-up period for ED based interventions. Importantly, future overviews, reviews and primary studies should maintain or direct their focus on patient-orientated outcomes and co-design to allow interventions to make the positive change required by patients and healthcare systems.

## Supplementary Information

Additional file 1.

Additional file 2.

Additional file 3.

Additional file 4.

Additional file 5.

## Data Availability

No datasets were generated or analysed during the current study.
