# Supplementary material for: Emergency department interventions and their effect on subsequent healthcare resource use after discharge: an overview of systematic reviews
Source: Scand J Trauma Resusc Emerg Med. 2025 May 1;33:76. doi: 10.1186/s13049-025-01377-4 (PMC12044817; doi:10.1186/s13049-025-01377-4)
Supplement: Supplementary file 1 — Additional file 1. [file 13049_2025_1377_MOESM1_ESM.docx]

Database: Ovid MEDLINE(R) ALL <1946 to February 05, 2021>

Search Strategy:

--------------------------------------------------------------------------------

1 shared Decision Making.mp. or exp Decision Making, Shared/ (9794)

2 Internet-Based Intervention/ or Intervention*.mp. (1107592)

3 exp Decision Making/ or joint decision making.mp. (207876)

4 patient involvement.mp. or Patient Participation/ (28380)

5 "patient engagement".mp. (3344)

6 clinical decision making.mp. or exp Clinical Decision-Making/ (28606)

7 decision support techniques/ or clinical decision rules/ (21343)

8 Patient Education as Topic/ (86318)

9 Pamphlets/ (3942)

10 (pamphlet* or leaflet*).mp. [mp=title, abstract, original title, name of substance word, subject heading word, floating sub-heading word, keyword heading word, organism supplementary concept word, protocol supplementary concept word, rare disease supplementary concept word, unique identifier, synonyms] (28005)

11 1 or 2 or 3 or 4 or 5 or 6 or 7 or 8 or 9 or 10 (1447994)

12 emergency service, hospital/ or trauma centers/ (82214)

13 Emergency Department*.mp. (99446)

14 emergency medicine.mp. or Emergency Medicine/ (26003)

15 emergency room*.mp. (20236)

16 emergency medical treatment*.mp. (363)

17 (accident and emergency).mp. (10450)

18 casualty department*.mp. (663)

19 emergency medical service*.mp. (49046)

20 Emergency Medical Services/ (43702)

21 emergency hospital*.mp. (1907)

22 emergency service*.mp. (79118)

23 12 or 13 or 14 or 15 or 16 or 17 or 18 or 19 or 20 or 21 or 22 (216319)

24 "continuity of patient care"/ or aftercare/ or patient discharge/ (56654)

25 discharge*.mp. (284132)

26 treatment outcome/ (1006510)

27 follow-up.mp. (1381605)

28 aftercare.mp. (11861)

29 Health Resources/ (13994)

30 (healthcare adj3 (use* or usage or using)).mp. (12582)

31 "downstream".mp. (162760)

32 (("General practice" or emergency or "111" or "999" or ambulance) adj3 (referral* or revisit* or reattend* or encounter or attend* or call or contact*)).mp. (8061)

33 24 or 25 or 26 or 27 or 28 or 29 or 30 or 31 or 32 (2510606)

34 11 and 23 and 33 (10945)

35 exp animals/ not humans/ (4785222)

36 34 not 35 (10919)

37 (((systematic or state-of-the-art or scoping or literature or umbrella) adj (review* or overview* or assessment*)) or "review* of reviews" or meta-analy* or metaanaly* or ((systematic or evidence) adj1 assess*) or "research evidence" or metasynthe* or meta-synthe*).tw. or exp Review Literature as Topic/ or exp Review/ or Meta-Analysis as Topic/ or Meta-Analysis/ or "systematic review"/ (2997307)

38 36 and 37 (1011)

39 limit 38 to english language (975)

***************************
