# Supplementary material for: Emergency department interventions and their effect on subsequent healthcare resource use after discharge: an overview of systematic reviews
Source: Scand J Trauma Resusc Emerg Med. 2025 May 1;33:76. doi: 10.1186/s13049-025-01377-4 (PMC12044817; doi:10.1186/s13049-025-01377-4)
Supplement: Supplementary file 2 — Additional file 2. [file 13049_2025_1377_MOESM2_ESM.docx]

| Question | Yes | Partial yes | No | N/A |
| --- | --- | --- | --- | --- |
| 9a For RCTs – Did the review authors use a satisfactory technique for assessing the risk of bias (RoB) in individual studies that were included in the review? | n=24 | n=2 | n=7 | n=5 |
| 9b For NRSI -Did the review authors use a satisfactory technique for assessing the risk of bias (RoB) in individual studies that were included in the review? | n=17 | n=4 | n=9 | n=8 |
| 12 If meta-analysis was performed, did the review authors assess the potential impact of RoB in individual studies on the results of the meta-analysis or other evidence synthesis? | n=11 | n=0 | n=2 | n=25 |
| 13 Did the review authors account for RoB in individual studies when interpreting/ discussing the results of the review? | n=27 | n=0 | n=11 | n=0 |
| 15 If they performed quantitative synthesis did the review authors carry out an adequate investigation of publication bias (small study bias) and discuss its likely impact on the results of the review? | n=7 | n=6 | n=0 | n=25 |
