# Supplementary material for: Emergency department interventions and their effect on subsequent healthcare resource use after discharge: an overview of systematic reviews
Source: Scand J Trauma Resusc Emerg Med. 2025 May 1;33:76. doi: 10.1186/s13049-025-01377-4 (PMC12044817; doi:10.1186/s13049-025-01377-4)
Supplement: Supplementary file 3 — Additional file 3. [file 13049_2025_1377_MOESM3_ESM.docx]

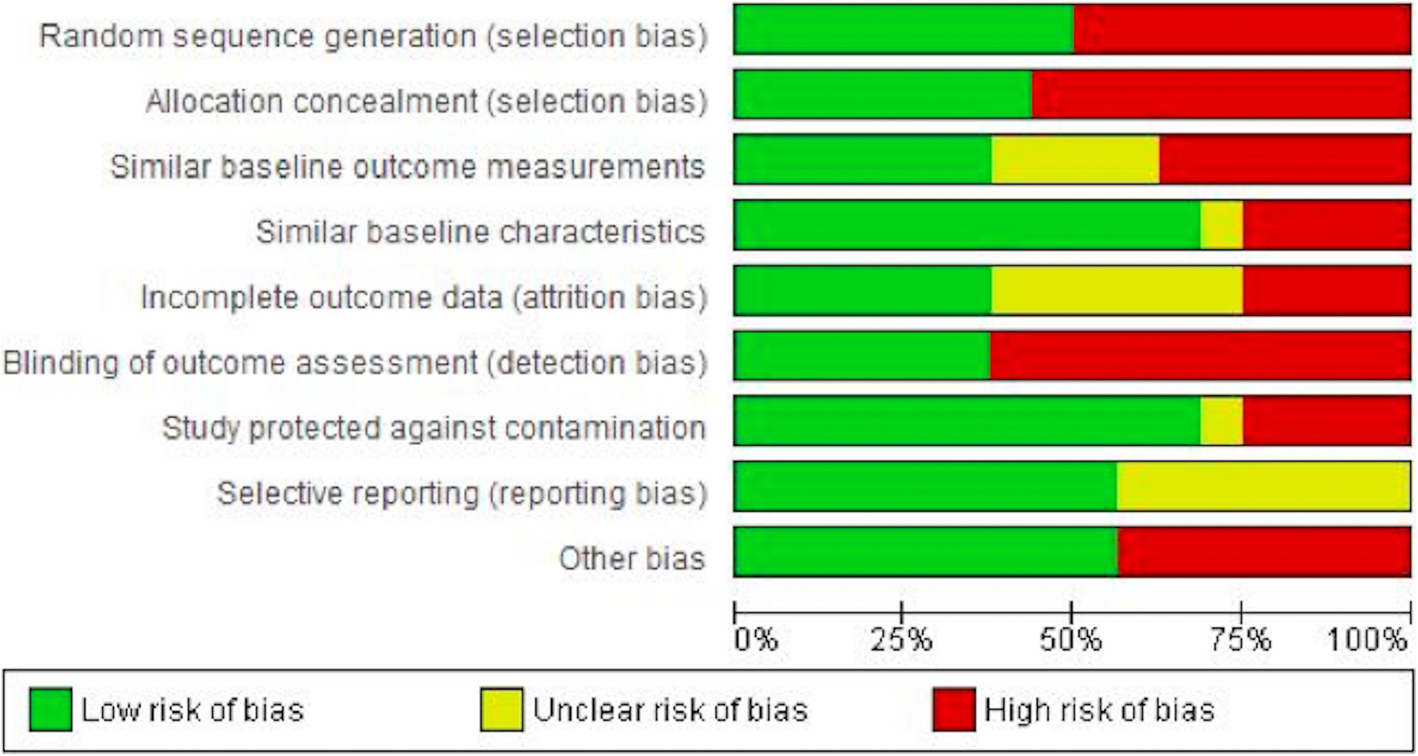


**Supplementary table 1.** Effectiveness of interventions to alleviate emergency department crowding by older adults: a systematic review. *BMC Emerg Med* **19,**69 (2019). Hesselink, G., Sir, Ö. & Schoon, Y. https://doi.org/10.1186/s12873-019-0288-4

Randomised

\
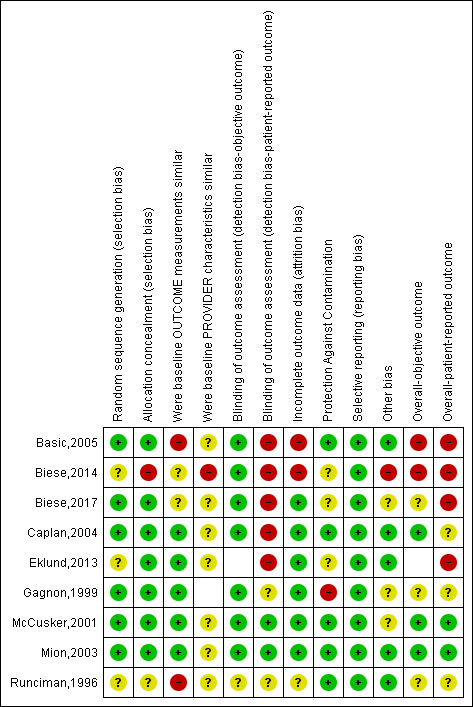


**Supplementary table 2.** Emergency Department Interventions for Older Adults: A Systematic Review. J Am Geriatr Soc, 67: 1516-1525. Hughes, J.M., Freiermuth, C.E., Shepherd-Banigan, M., Ragsdale, L., Eucker, S.A., Goldstein, K., Hastings, S.N., Rodriguez, R.L., Fulton, J., Ramos, K., Tabriz, A.A., Gordon, A.M., Gierisch, J.M., Kosinski, A. and Williams, J.W., Jr (2019), <https://doi.org/10.1111/jgs.15854>

Key: White indicates items that were not applicable. Green/positive indicates items that were judged low risk of bias. Yellow/question mark indicates items that were judged unclear ROB. Red/negative indicates items that were judged high risk of bias.

Non randomised

**
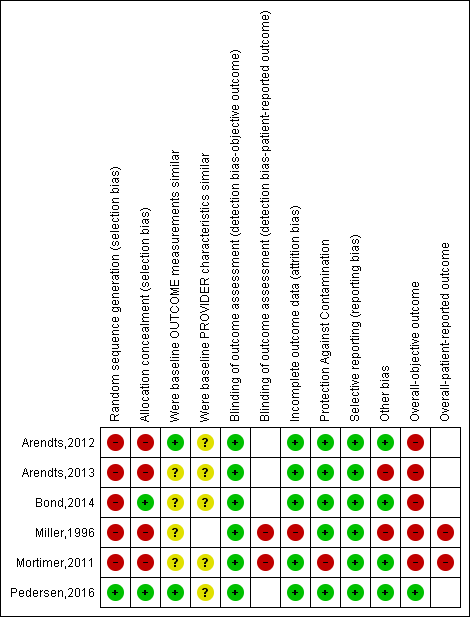
**

**Supplementary table 3.** Emergency Department Interventions for Older Adults: A Systematic Review. J Am Geriatr Soc, 67: 1516-1525. Hughes, J.M., Freiermuth, C.E., Shepherd-Banigan, M., Ragsdale, L., Eucker, S.A., Goldstein, K., Hastings, S.N., Rodriguez, R.L., Fulton, J., Ramos, K., Tabriz, A.A., Gordon, A.M., Gierisch, J.M., Kosinski, A. and Williams, J.W., Jr (2019), <https://doi.org/10.1111/jgs.15854>

Key: White indicates items that were not applicable. Green/positive indicates items that were judged low ROB. Yellow/question mark indicates items that were judged unclear ROB. Red/negative indicates items that were judged high ROB.

|  | Goldstein | CT-STAT | ACRIN-PA | ROMICAT II | Median |
| --- | --- | --- | --- | --- | --- |
| **Jadad criteria** |  |  |  |  |  |
| Study described as "randomized?" | 1 | 1 | 1 | 1 | 1 |
| Randomization appropriate? | 1 | 1 | 1 | 1 | 1 |
| Study described as "double blinded?" | 0 | 0 | 0 | 0 | 0 |
| Blinding appropriate? | 0 | 0 | 0 | 0 | 0 |
| Adequate description of withdrawals? | 1 | 1 | 1 | 1 | 1 |
| Statistical methods adequately described? | 1 | 1 | 1 | 1 | 1 |
| Clear description of inclusion and exclusion criteria? | 1 | 1 | 1 | 1 | 1 |
| Method of assessing for adverse effects described? | 1 | 1 | 1 | 1 | 1 |
| Median Jadad Score | 6 | 6 | 6 | 6 | 6 |
|  |  |  |  |  |  |
| **Cochrane Risk of Bias** |  |  |  |  |  |
| Was the allocation sequence adequately generated? | Low | Low | Low | Low | Low |
| Was allocation adequately concealed? | Low | Low | Low | Low | Low |
| Was knowledge of the allocated intervention prevented? | Unclear | Unclear | Unclear | Unclear | Unclear |
| Were incomplete outcome data adequately addressed? | Low | Low | Low | Low | Low |
| Free of other problems that risk bias? | Unclear | Unclear | Low | Low | Unclear |

**Supplementary Table 4.** Results of quality of reporting assessment using Jadad scale and risk of bias using the Cochrane tool for assessment of risk of bias.Hulten, E. *et al.* Outcomes After Coronary Computed Tomography Angiography in the Emergency Department: A Systematic Review and Meta-Analysis of Randomized, Controlled Trials. *Journal of the American College of Cardiology* 61, 880–892 (2013).

**Table S9 Risk of bias**

|  | **Random sequence generation** | **Allocation sequence concealment** | **Blinding of participants and personnel** | **Blinding of outcome assessment** | **Incomplete outcome data** | **Selective outcome reporting** | **Other potential sources of bias** |
| --- | --- | --- | --- | --- | --- | --- | --- |
| **Active contact and follow-up group (Intensive care plus outreach)** | | | | | | | |
| van der Sande et al., 1997[^4^](#_ENREF_4) | Low | Low | Unclear | High | Unclear | Low | High |
| Hatcher et al., 2015[^7^](#_ENREF_7) | Low | Low | Unclear | Unclear | Low | Low | Unclear |
| **Active contact and follow-up group (Brief intervention and contact)** | | | | | | | |
| Hassanian-Moghaddam et al., 2011[^15^](#_ENREF_15); 2015[^16^](#_ENREF_16) | Low | Low | Unclear | High | Low | Low | Low |
| **Active contact and follow-up group (Composite of letter/postcard and telephone)** | | | | | | | |
| Kapur et al., 2013[^19^](#_ENREF_19) | Low | Low | Unclear | Low | Low | High | High |

|  | **Random sequence generation** | **Allocation sequence concealment** | **Blinding of participants and personnel** | **Blinding of outcome assessment** | **Incomplete outcome data** | **Selective outcome reporting** | **Other potential sources of bias** |
| --- | --- | --- | --- | --- | --- | --- | --- |
| **Psychotherapy group** | | | | | | | |
| Gibbons et al., 1978[^20^](#_ENREF_20) | High | High | Unclear | Low | High | High | Unclear |
| Guthrie et al., 2001[^23^](#_ENREF_23) | Low | Unclear | Unclear | Low | Low | Low | High |

**Supplementary table 5.**Active contact and follow-up interventions to prevent repeat suicide attempts during high-risk periods among patients admitted to emergency departments for suicidal behavior: a systematic review and meta-analysis. *BMC Psychiatry* **19,**44 (2019). Inagaki, M., Kawashima, Y., Yonemoto, N. *et al.* <https://doi.org/10.1186/s12888-019-2017-7>
Abbreviations: Low, low risk of bias (plausible bias unlikely to seriously alter the results); Unclear, unclear risk of bias (plausible bias that raises some doubts about the results); High, high risk of bias (plausible bias that seriously weakens confidence in the result


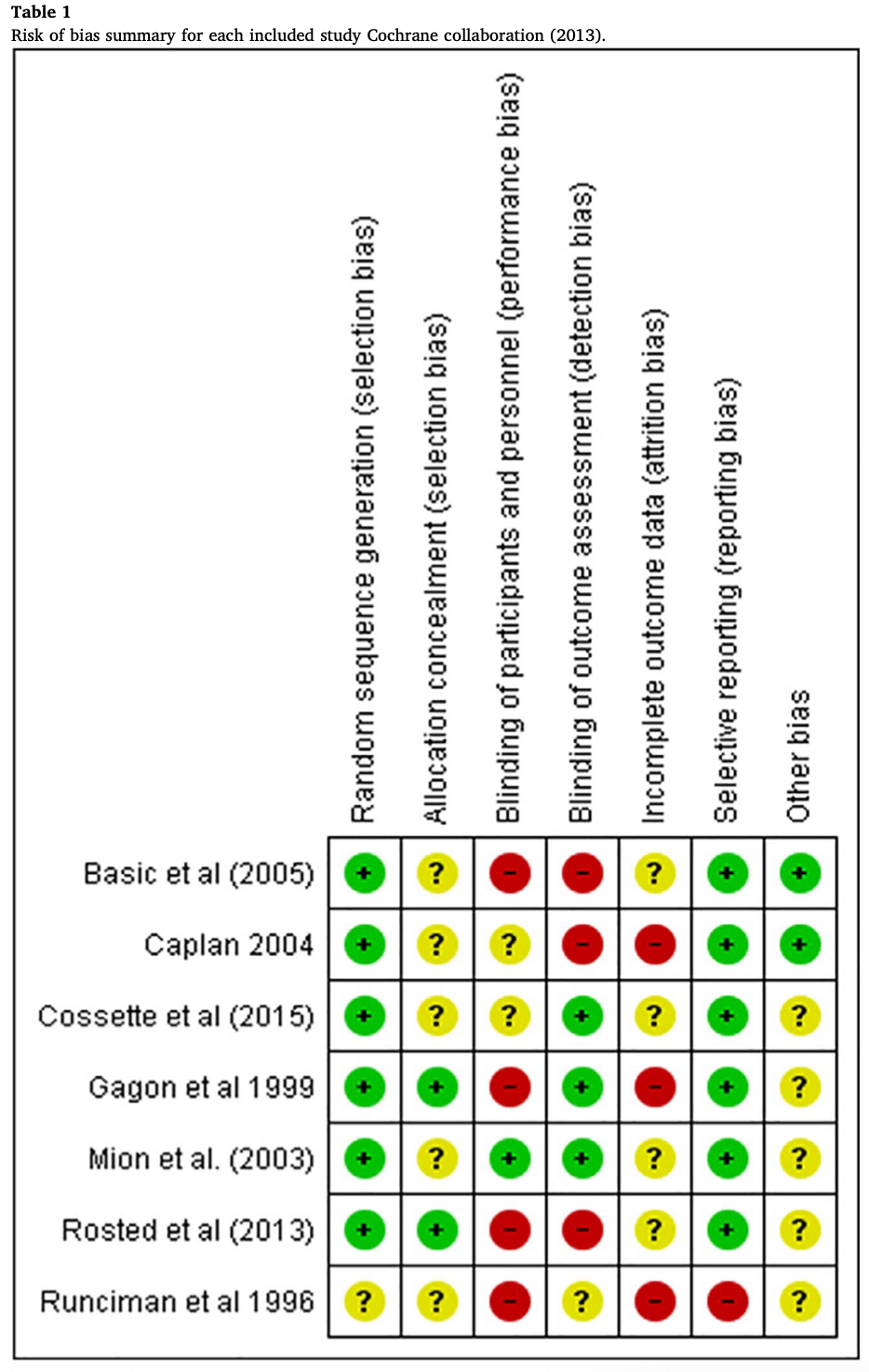


**Supplementary table 6.** Malik, M. *et al.* (2018) ‘The impact of geriatric focused nurse assessment and intervention in the emergency department: A systematic review.’, *International Emergency Nursing*. Emergency Department, St. James’s Hospital, James’s Street, Dublin 8, Ireland: Elsevier B.V., 37, pp. 52–60. doi: 10.1016/j.ienj.2018.01.008.


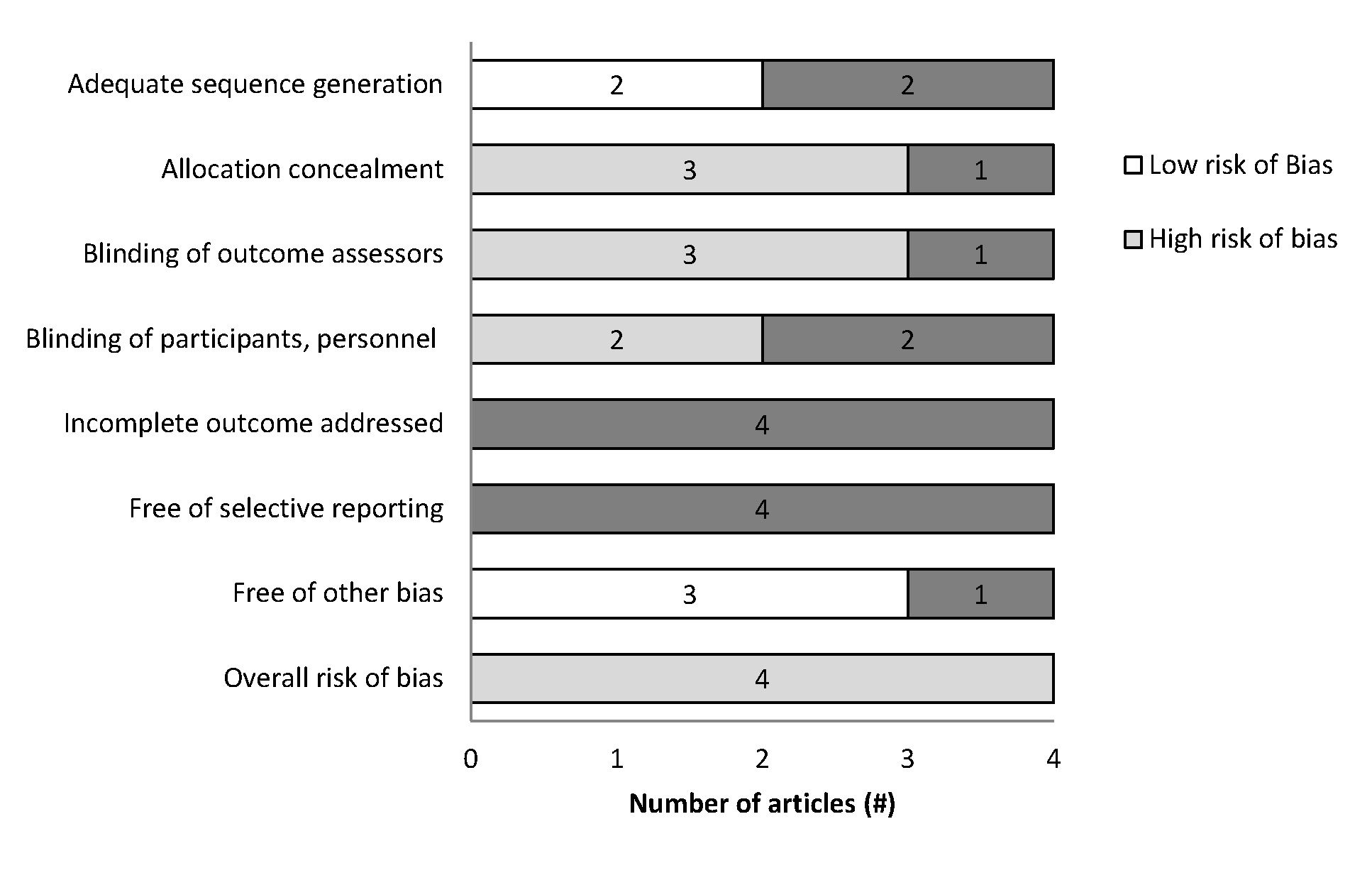


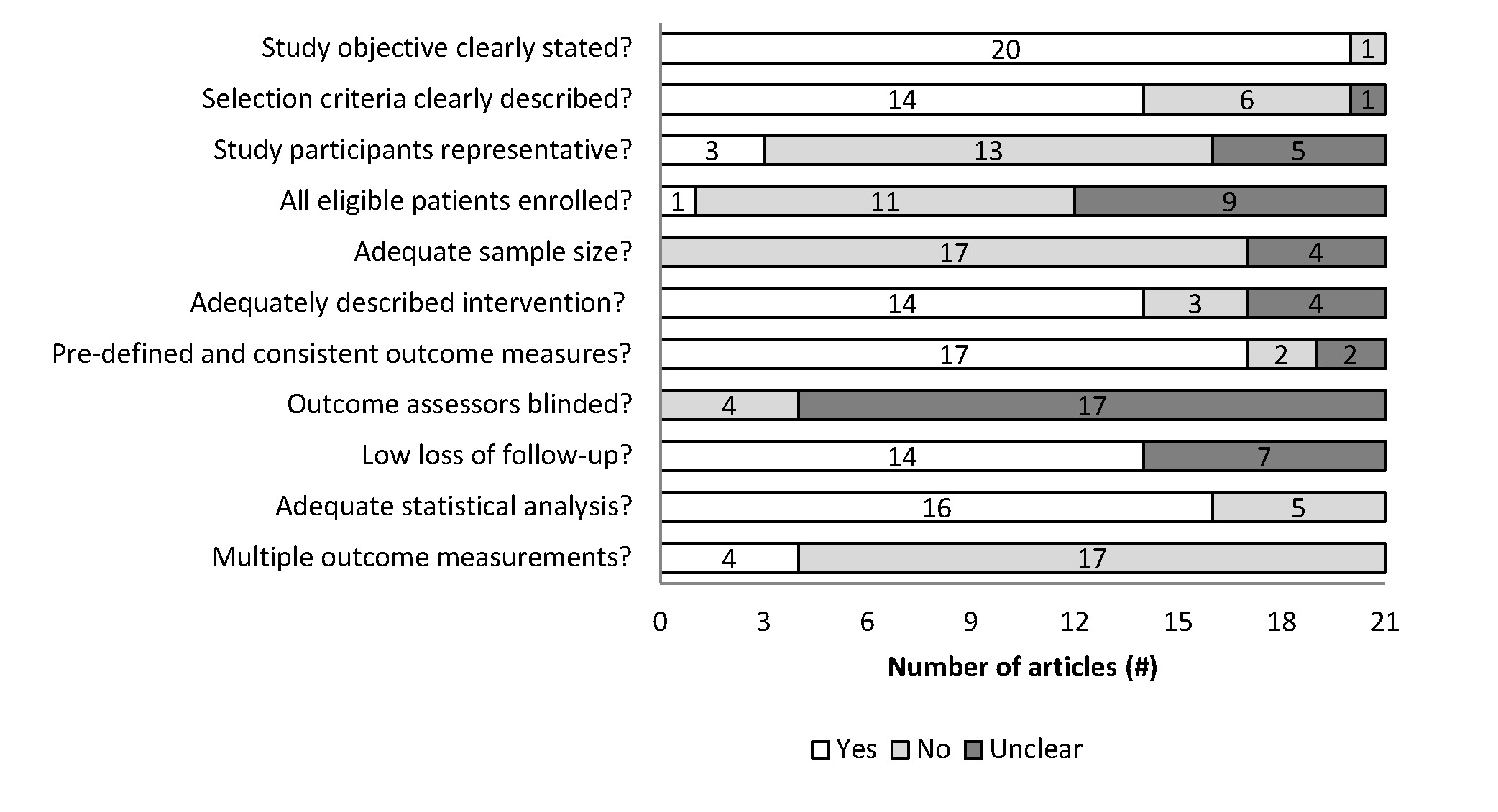


# Supplementary table 7. Effectiveness of Interventions to Decrease Emergency Department Visits by Adult Frequent Users: A Systematic Review [Jessica Moe MD](https://onlinelibrary.wiley.com/action/doSearch?ContribAuthorRaw=Moe%2C+Jessica), [Scott W. Kirkland MSc](https://onlinelibrary.wiley.com/action/doSearch?ContribAuthorRaw=Kirkland%2C+Scott+W), [Erin Rawe MD](https://onlinelibrary.wiley.com/action/doSearch?ContribAuthorRaw=Rawe%2C+Erin), [Maria B. Ospina MSc, PhD](https://onlinelibrary.wiley.com/action/doSearch?ContribAuthorRaw=Ospina%2C+Maria+B), [Ben Vandermeer MSc](https://onlinelibrary.wiley.com/action/doSearch?ContribAuthorRaw=Vandermeer%2C+Ben), [Sandy Campbell MLS](https://onlinelibrary.wiley.com/action/doSearch?ContribAuthorRaw=Campbell%2C+Sandy), [Brian H. Rowe MD](https://onlinelibrary.wiley.com/action/doSearch?ContribAuthorRaw=Rowe%2C+Brian+H)[… See fewer authors](https://onlinelibrary.wiley.com/doi/10.1111/acem.13060)First published: 30 July 2016 <https://doi.org/10.1111/acem.13060>


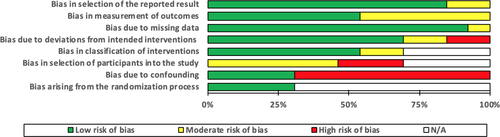


**Supplementary table 8.** Summary of the study quality assessments using the RoB 2.0 (RCTs) and ROBINS-I (NCBAS) tools. The Characteristics and Effectiveness of Interventions for Frequent Emergency Department Utilizing Patients With Chronic Noncancer Pain: A Systematic Review [Charles K. Wong MD, CCFP-EM](https://onlinelibrary.wiley.com/action/doSearch?ContribAuthorRaw=Wong%2C+Charles+K), [Connor M. O'Rielly](https://onlinelibrary.wiley.com/action/doSearch?ContribAuthorRaw=O%27Rielly%2C+Connor+M), [Braden D. Teitge MD, CCFP](https://onlinelibrary.wiley.com/action/doSearch?ContribAuthorRaw=Teitge%2C+Braden+D)[**et**](https://onlinelibrary.wiley.com/doi/10.1111/acem.13934) **al** First published: 06 February 2020

<https://doi.org/10.1111/acem.13934>


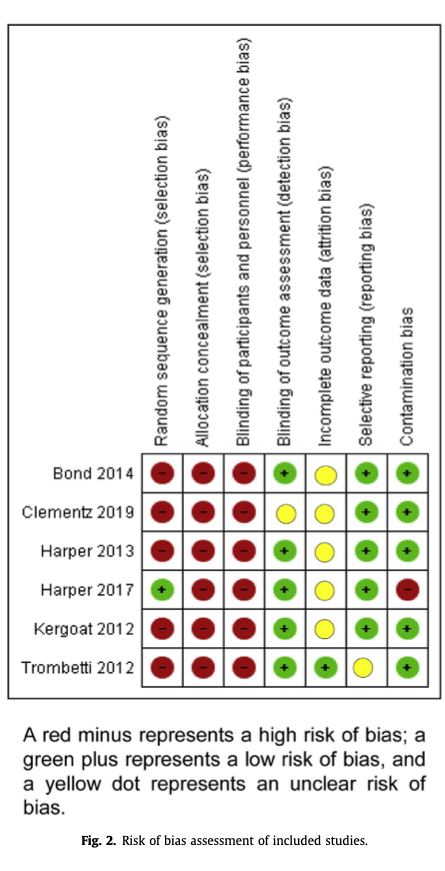


**Supplementary table 9.** Readmissions of Elder Patients Presenting to Hospital for a Fall (RELIEF): A Systematic Review. Carole Ratsimbazafy MSca,*, Camille Schwab PharmD, MSca,b, Agnès Dechartres MD, PhD c, Christine Fernandez PharmD, PhD a, b, Patrick Hindlet PharmD, PhDa,b

***Non-randomised studies***

Were the participants included in any comparisons similar?

Is it clear in the study what is the ‘cause’ and what is the ‘effect’ (i.e. there is no confusion about which variable comes first)?

Were the outcomes of participants included in any comparisons measured in the same way?

Were outcomes measured in a reliable way?

Was appropriate statistical analysis used?

Was follow up complete and if not, were differences between groups in terms of their follow up adequately described and analysed?

Were there multiple measurements of the outcome both pre and post the intervention/exposure?

Was there a control group?

Were the participants receiving similar care, other than the exposure or intervention of interest?

Crilly 2011 [26]

Fan 2016 [25]

***Case series study***

Was statistical analysis appropriate?

Was there clear reporting of the presenting site(s)/clinic(s) demographic information?

Were the outcomes or follow up results of cases clearly reported?

Was there clear reporting of clinical information of the participants?

Was there clear reporting of the demographics of the participants in the study?

Did the case series have complete inclusion of participants?

Were valid methods used for identification of the condition for all participants included in the case series?

Was the condition measured in a standard, reliable way for all participants included in the case series?

Were there clear criteria for inclusion in the case series?

Did the case series have consecutive inclusion of participants?

Lau 2013 [22]

**Supplementary table 10.** Santosaputri, E., Laver, K. and To, T. (2019) ‘Efficacy of interventions led by staff with geriatrics expertise in reducing hospitalisation in nursing home residents: A systematic review.’, *Australasian journal on ageing*. Australia, 38(1), pp. 5–14. doi: <https://dx.doi.org/10.1111/ajag.12593>. Key: Red- High risk; Yellow – Unclear risk; Green – Low risk; White – non-applicable.

Unclear risk

High risk

Low risk

Not applicable


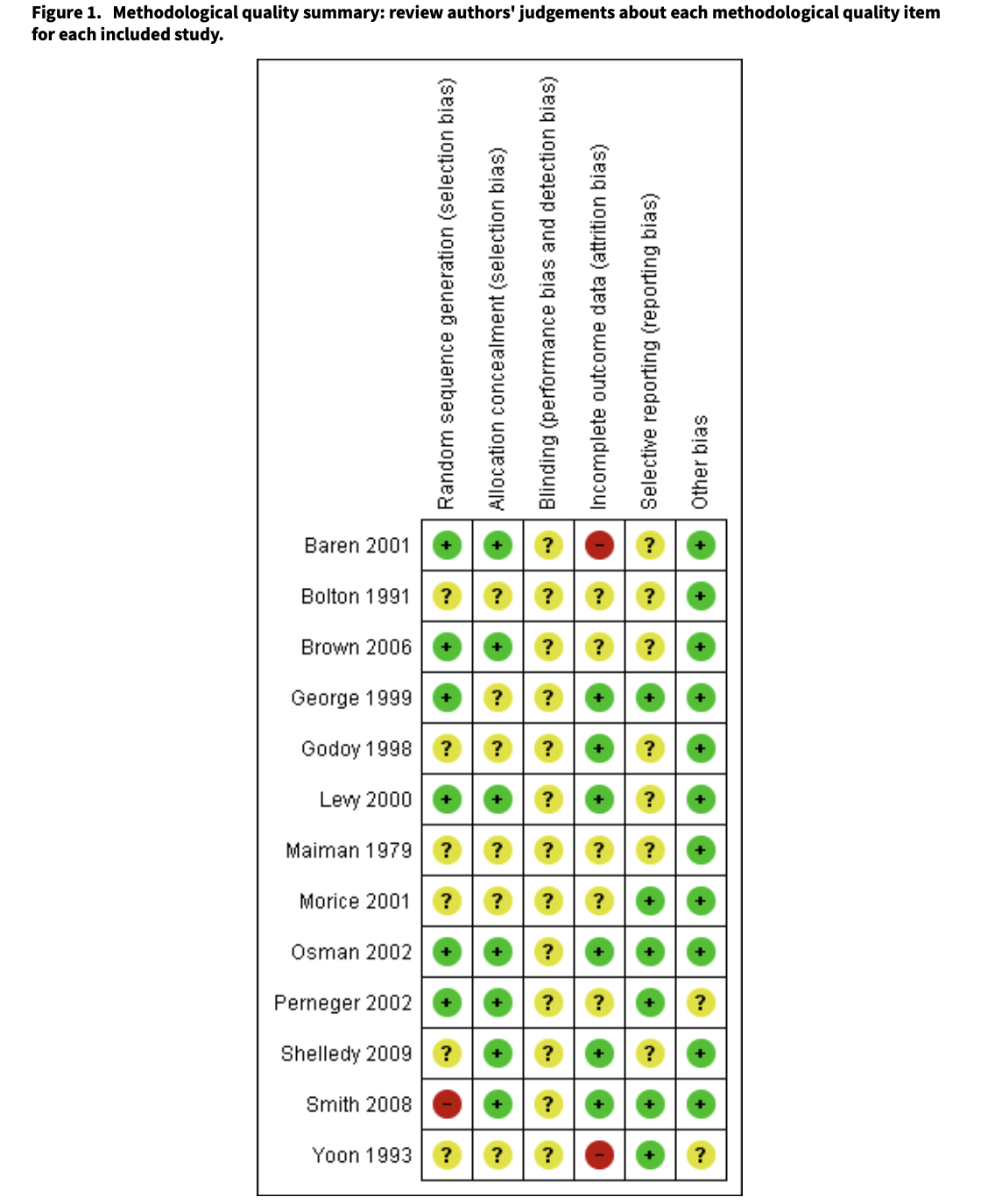


**Supplementary table 10.** Education interventions for adults who attend the emergency room for acute asthma (Review) Tapp S, Lasserson TJ, Rowe Bh. Education interventions for adults who attend the emergency room for acute asthma. Cochrane Database Syst Rev. 2007 Jul 18;(3):CD003000. doi: 10.1002/14651858.CD003000.pub2. PMID: 17636712.

Only ones included =

Only ones included =

Only ones included =

Only ones included =


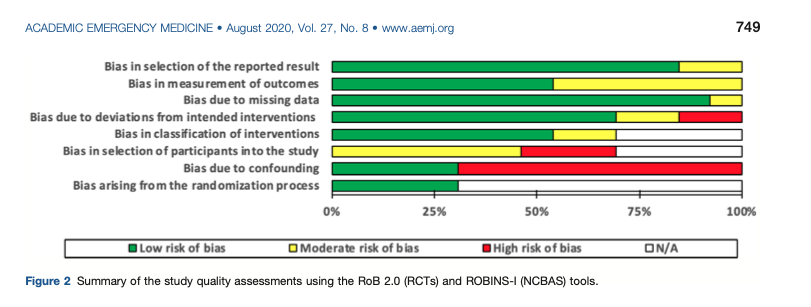


**Supplementary table 11.** Wong CK, O'Rielly CM, Teitge BD, Sutherland RL, Farquharson S, Ghosh M, Robertson HL, Lang E. The Characteristics and Effectiveness of Interventions for Frequent Emergency Department Utilizing Patients With Chronic Noncancer Pain: A Systematic Review. Acad Emerg Med. 2020 Aug;27(8):742-752. doi: 10.1111/acem.13934. Epub 2020 Mar 9. PMID: 32030836.


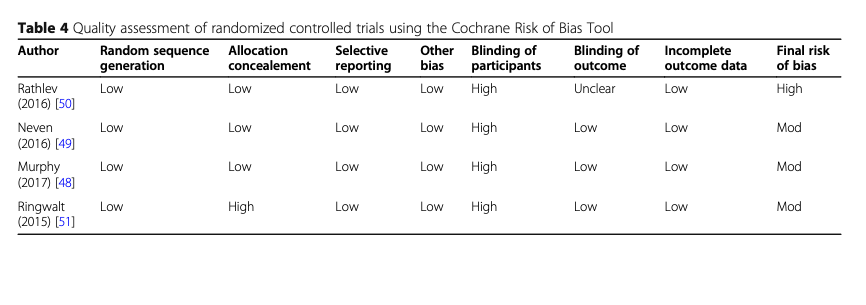

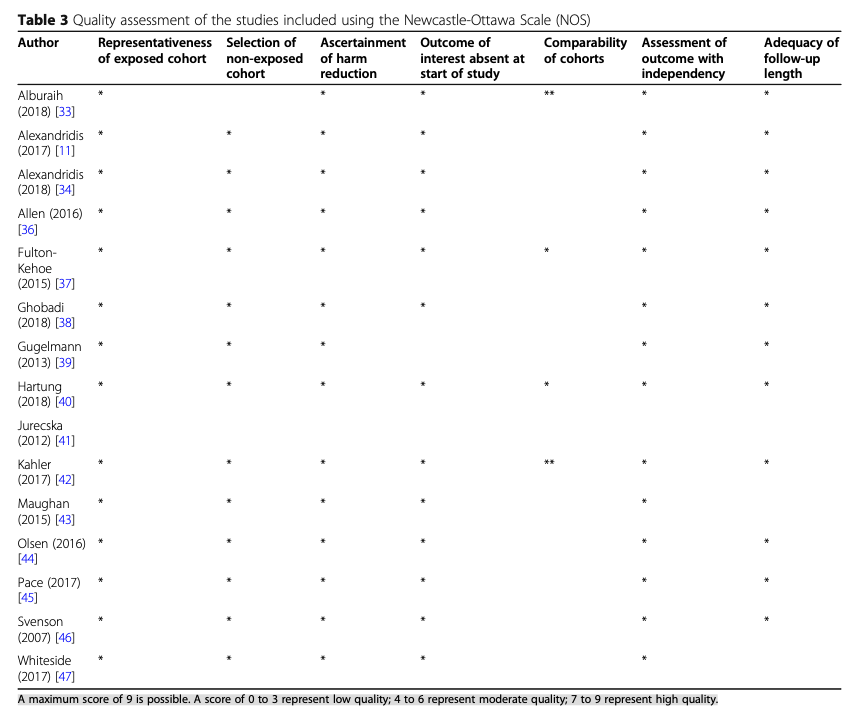


**Supplementary tables 12.** Association between supportive interventions and healthcare utilization and outcomes in patients on long-term prescribed opioid therapy presenting to acute healthcare settings: a systematic review and meta-analysis Jean Deschamps1* , James Gilbertson2, Sebastian Straube3, Kathryn Dong4, Frank P. MacMaster5, Christina Korownyk6, Lori Montgomery7, Ryan Mahaffey8, James Downar9, Hance Clarke10,11, John Muscedere12, Katherine Rittenbach13,14,15, Robin Featherstone16, Meghan Sebastianski17, Ben Vandermeer16, Deborah Lynam18, Ryan Magnussen19, Sean M. Bagshaw1 and Oleksa G. Rewa1

**Appendix III: Risk of bias results for included studies**

| **JBI Critical Appraisal Checklist for Randomised Controlled Trials^a^ (n = 12)** | | | | | | | | | | | | | |
| --- | --- | --- | --- | --- | --- | --- | --- | --- | --- | --- | --- | --- | --- |
| Study | Q1 | Q2 | Q3 | Q4 | Q5 | Q6 | Q7 | Q8 | Q9 | Q10 | Q11 | Q12 | Q13 |
| Chu (2017) | Y | Y | Y | U | Y | Y | U | Y | Y | Y | Y | Y | Y |
| ^b^Cosette, Vadeboncoeur et al. (2015) | Y | Y | Y | N | N | Y | Y | Y | Y | Y | Y | Y | Y |
| ^b^Cosette, Frasure et al. (2015) | Y | Y | Y | N | N | Y | Y | Y | Y | Y | Y | Y | Y |
| Goldberg (2020) | Y | Y | Y | U | N | U | Y | Y | Y | Y | Y | Y | Y |
| Harper (2017) | N | N | N | Y | N | Y | Y | Y | Y | Y | Y | Y | Y |
| Hayes (1998) | N | Y | Y | U | Y | N | Y | Y | Y | Y | Y | Y | N |
| Hendriksen (2001) | Y | Y | Y | N | U | N | Y | Y | Y | Y | Y | Y | Y |
| ^c^McCusker, Verdon (2001) | Y | U | N | U | N | U | Y | Y | Y | Y | N | U | U |
| ^c^McCusker, Jacobs (2003) | Y | U | N | U | N | U | Y | Y | Y | Y | N | U | U |
| ^McCusker, Dendukuri (2003) | Y | U | N | U | N | U | Y | Y | Y | Y | N | U | U |
| Mion (2003) | Y | Y | Y | N | U | Y | Y | Y | Y | Y | Y | Y | Y |
| Ong (2018) | Y | Y | Y | N | N | N | Y | Y | Y | Y | Y | Y | Y |
| Pedersen (2016) | N | U | Y | U | N | U | Y | Y | Y | Y | Y | Y | U |

^a^Y = Yes; N = No; U = Unclear; N/A = not applicable JBI Critical appraisal checklist for Randomized Controlled Trials: Q1 = Was true randomization used for assignment of participants to treatment groups?; Q2 = Was allocation to treatment groups concealed?; Q3 = Were treatment groups similar at baseline?; Q4 = Were participants blind to treatment assignment?; Q5 = Were those delivering treatment blind to treatment assignment?; Q6 = Were outcome assessors blind to treatment assignment?; Q7 = Were treatment groups treated identically other than the intervention of interest?; Q8 = Was follow-up complete and if not, were differences between groups in terms of their follow-up adequately described and analyzed?; Q9 = Were participants analyzed in the groups to which they were randomized?; Q10 = Were outcomes measured in the same way for treatment groups?; Q11 = Were outcomes measured in a reliable way?; Q12 = Was appropriate statistical analysis used?; Q13 = Was the trial design appropriate, and any deviations from the standard RCT design (individual randomization, parallel groups) accounted for in the conduct and analysis of the trial?^b^ Cosette et al., ^c^McCusker et al. same RCT(s)

| **JBI Critical Appraisal Checklist for Quasi Experimental studies^d^ (n = 11)** | | | | | | | | | | |
| --- | --- | --- | --- | --- | --- | --- | --- | --- | --- | --- |
| Study | Q1 | Q2 | Q3 | Q4 | Q5 | Q6 | Q7 | Q8 | Q9 |  |
| Arendts (2013) | U | Y | Y | Y | N/A | U | Y | Y | Y |  |
| Bond (2014) | Y | Y | U | Y | Y | Y | Y | Y | Y |  |
| Capp (2017) | Y | U | U | Y | Y | U | Y | Y | U |  |
| Conroy (2014) | Y | Y | Y | Y | Y | Y | Y | Y | Y |  |
| Foo (2012) | Y | Y | Y | Y | Y | Y | Y | Y | Y |  |
| Guttman (2004) | Y | Y | Y | Y | Y | Y | Y | U | Y |  |
| Hardy (2001) | Y | U | U | Y | N | U | Y | Y | Y |  |
| Heeren (2019) | Y | N | U | N | Y | Y | Y | Y | Y |  |
| Miller (1996) | Y | Y | Y | Y | Y | Y | Y | N | N |  |
| Mion (2001) | Y | N | N | Y | Y | N | N | Y | U |  |
| Stevens (2015) | Y | U | U | Y | Y | U | Y | Y | Y |  |
| Stevens (2017) | Y | U | U | Y | Y | U | Y | Y | Y |  |

^d^Y = Yes; N = No; U = Unclear, N/A = not applicable; Q1 = Is it clear in the study what is the ‘cause’ and what is the ‘effect’ (i.e. there is no confusion about which variable comes first)?; Q2 = Were the participants included in any comparisons similar?; Q3 = Were the participants included in any comparisons receiving similar treatment/care, other than the exposure or intervention of interest?; Q4 = Was there a control group?; Q5 = Were there multiple measurements of the outcome both pre and post the intervention/exposure?; Q6 = Was follow up complete and if not, were differences between groups in terms of their follow up adequately described and analyzed?; Q7 = Were the outcomes of participants included in any comparisons measured in the same way?; Q8 = Were outcomes measured in a reliable way?; Q9 = Was appropriate statistical analysis used?

**Supplementary tables 13.** Interventions for the discharge of older people to their home from the emergency department: a systematic review. Rosalind Elliotta,b,∗, Joy Meic, Nicola Wormleatond, Margaret Frya,b

| **Study** | | | | | | **Quality assessment criteria** | | | | | |  | | | | | |
| --- | --- | --- | --- | --- | --- | --- | --- | --- | --- | --- | --- | --- | --- | --- | --- | --- | --- |
|  | **A1** | **A2** | **B1** | **B2** | **B3** | | **B4** | **C1** | **C2** | **D1** | **D2** | | **E1** | **E2** | **F1** | **F2** | **OVERALL** |
| Chu 2017 | 1 | 2 | 1 | 2 | 2 | | 2 | 2 | - | 2 | 3 | | 1 | 1 | 1 | 1 | Strong |
| Close 1999 | 1 | 2 | 1 | 2 | 2 | | 2 | 2 | - | 3 | 1 | | 3 | 3 | 1 | 2 | Moderate |
| Davison 2005 | 1 | 3 | 1 | 2 | 2 | | 2 | 2 | - | 1 | 3 | | 1 | 1 | 1 | 1 | Moderate |
| Harper 2017 | 1 | 1 | 2 | 2 | 2 | | 2 | 1 | 1 | 2 | 1 | | 1 | 1 | 1 | 1 | Strong |
| Lightbody 2002 | 2 | 3 | 1 | 2 | 2 | | 2 | 2 | - | 3 | 3 | | 1 | 1 | 1 | 1 | Moderate |
| Russell 2010 | 2 | 1 | 1 | 2 | 2 | | 2 | 2 | - | 2 | 3 | | 3 | 3 | 1 | 1 | Moderate |

**Supplementary table 14.** Quality scores for the included studies (Cochrane as per the Quality Assessment Tool for Quantitative Studies, Effective Public Health Practice Project 2007.43 ) Providing fall prevention services in the emergency department: Is it effective? A systematic review and meta-analysis Kristie J. Harper1 | Glenn Arendts2

|  | NIH Quality Grade | Newcastle-Ottawa Risk of Bias |
| --- | --- | --- |
| Dumkow18 | Fair | Moderate |
| Dumkow19 | Good | Moderate |
| Ellena20 | Good | Moderate |
| Giruzzi22 | Fair | Moderate |
| Olson30 | Fair | Moderate |
| Randolph31 | Fair | High |
| Santiago32 | Fair | Moderate |
| Shealy33 | Fair | Moderate |
| Stoll34 | Fair | Moderate |
| Zimmerman35 | Good | Moderate |

**Supplementary table 15.** Risk of bias table 2. Study Author. Impact of Pharmacist-Led Antimicrobial Stewardship on Appropriate Antibiotic Prescribing in the Emergency Department: A Systematic Review and Meta-Analysis Kirstin Kooda, PharmD*; Elizabeth Canterbury, PharmD; Fernanda Bellolio, MD, MS

| **Author, date,**  **country,**  **setting** | **Random sequence generation** | **Allocation concealment** | **Blinding of participants/ personnel** | **Blinding of outcome assessment** | **Incomplete outcome data** | **Selective reporting** | **Other bias** |
| --- | --- | --- | --- | --- | --- | --- | --- |
| Biese et al,  2014, USA, academic center ED | Blinded, block randomiza-  tion | Blinded, using marbles in a bag | Patients were blinded.  Nurse who did intervention was not blinded. Telephone calls were scripted. | Research assistants who did data collection phone calls were blinded for randomization, but might have known who was in the control group, as they had to perform a mental screening test only in control group patients, whereas other patients were tested earlier. | Incomplete data of 6 (4.5%) patients. 37 (23.6%) eligible patients were not included, due to refusal or not being reached.  Unclear whether patients were analyzed according to intention to treat. | Research protocol published in advance. Methods are followed and expected outcomes reported as planned. | Single center  Most outcome data were self-reported by patients.  Unknown how often the nurse helped patients making follow-up appointments.  Exclusion of potentially important individuals: patients not instructed to seek outpatient follow-up, patients visiting the ED in the weekend and patients and caregivers who did not pass the mental cognition screening examination. |
| Biese et al,  2018, USA, academic center ED | Randomiza-  tion with randomly generated block sizes of 4, 6 and 8. | Blinded, using a random sequence generator, imbedded in the computer program | Patients were blinded.  Nurses who did intervention were not blinded. Calls were scripted, recorded and reviewed to ensure adherence to the scripts. | Investigators were blinded for randomization. Unclear whether nurses who did data collection phone calls after 30 days were blinded for randomization.  Statistician was not blinded. | Loss to follow-up was limited (<1%), equally divided over groups and reasons for missing data were described.  Many eligible patients not included, due to decline or not being reached. | Research protocol published in advance. Methods are followed and expected outcomes reported as planned. | Single center  Many outcomes were self-reported by patients.  Participation bias not excluded as number of hospital admissions in both groups lower than expected.  After all underpowered study due to lower number of hospital admissions than predicted.  Patients and caregivers who did not pass the mental cognition screening examination were excluded. |

# Supplementary table 16. The effect of a telephone follow-up call for older patients, discharged home from the emergency department on health-related outcomes: a systematic review of controlled studies, [Merel van Loon-van Gaalen](https://intjem.biomedcentral.com/articles/10.1186/s12245-021-00336-x#auth-Merel-van_Loon_van_Gaalen) Cristina Villa-Roel MD, MSc, PhD, Britt Voaklander Bkin(c), Maria B. Ospina MSc, PhD, Taylor Nikel MD(c), Sandra Campbell MLIS & Brian H. Rowe MD, MSc, CCFP(EM), FCCP (2017): Effectiveness of written action plans for acute asthma: A systematic review, Journal of Asthma, DOI: 10.1080/02770903.2017.1318142 Risk of bias assessment: The risk of bias was low in two trials.(17, 18) One trial that used a non-random sequence generation and failed to describe allocation concealment and blinding of outcome assessors was rated as at high risk of bias.(16) Key: ED: Emergency department


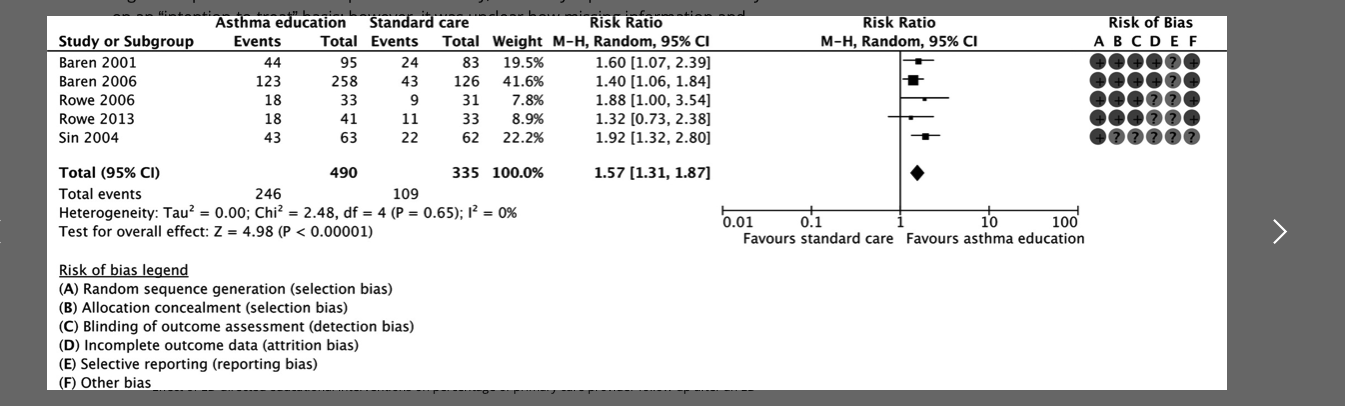


# Supplementary table 17. Effectiveness of Educational Interventions to Increase Primary Care Follow-up for Adults Seen in the Emergency Department for Acute Asthma: A Systematic Review and Meta-analysis, [Cristina Villa-Roel MD, MSc](https://onlinelibrary.wiley.com/action/doSearch?ContribAuthorRaw=Villa-Roel%2C+Cristina), [Taylor Nikel](https://onlinelibrary.wiley.com/action/doSearch?ContribAuthorRaw=Nikel%2C+Taylor), [Maria Ospina PhD](https://onlinelibrary.wiley.com/action/doSearch?ContribAuthorRaw=Ospina%2C+Maria), [Britt Voaklander](https://onlinelibrary.wiley.com/action/doSearch?ContribAuthorRaw=Voaklander%2C+Britt)

The overall risk of bias of all five included studies was rated as unclear. Sequence generation and allocation concealment were judged to be at *low* risk of bias in all but one of the included studies.[29](https://onlinelibrary.wiley.com/doi/10.1111/acem.12837#acem12837-bib-0029) This study reported the randomization of weeks following a “time-series method”; however, no details were provided with regard to the methods used to generate this allocation sequence or to prevent this sequence from being predicted in advance of enrolment. Four studies reported having outcome assessors blinded to the study interventions[25](https://onlinelibrary.wiley.com/doi/10.1111/acem.12837#acem12837-bib-0025)-[28](https://onlinelibrary.wiley.com/doi/10.1111/acem.12837#acem12837-bib-0028) and two were free of incomplete outcome data.[27](https://onlinelibrary.wiley.com/doi/10.1111/acem.12837#acem12837-bib-0027), [28](https://onlinelibrary.wiley.com/doi/10.1111/acem.12837#acem12837-bib-0028) All studies were judged to be at unclear risk of bias for selective outcome reporting due to the lack of registered protocols or full-text publication. Finally, one study reported data to be analysed on an “intention-to-treat” basis; however, it was unclear how missing information and dropouts were handled in the analyses.[29](https://onlinelibrary.wiley.com/doi/10.1111/acem.12837#acem12837-bib-0029) The principal investigator of two studies provided details on study methods and original data;[25](https://onlinelibrary.wiley.com/doi/10.1111/acem.12837#acem12837-bib-0025), [26](https://onlinelibrary.wiley.com/doi/10.1111/acem.12837#acem12837-bib-0026) the risk of bias assessment of these studies was based on the information provided by the study author and the available publications (abstracts; please see Figure [2](https://onlinelibrary.wiley.com/doi/10.1111/acem.12837#acem12837-fig-0002)).

| Before–After Quality Assessment | | |
| --- | --- | --- |
|  | **Haig 29** | **Min 27** |
| Selection | | |
| Representative postintervention group | Y | Y |
| Representative preintervention group | Y | Y |
| Pre- and postintervention groups from the same source | Y | Y |
| Comparability | | |
| Comparable design or analysis of both groups | U | U |
| Assessment of outcome | | |
| Valid assessment of outcome(s) | Y | Y |
| Reliable/accurate assessment of outcome(s) | U | U |
| Same method of outcome assessment for both groups | Y | U |
| Intervention | | |
| Intervention at a clearly defined point in time | N | Y |
| Intervention clearly described | Y | Y |
| Data collected during a similar timeframe | U | N |

# Supplementary table 18. Effectiveness of Interventions to Decrease Image Ordering for Low Back Pain Presentations in the Emergency Department: A Systematic Review. [Chaocheng Liu BSc](https://onlinelibrary.wiley.com/action/doSearch?ContribAuthorRaw=Liu%2C+Chaocheng), [Shashwat Desai BSc](https://onlinelibrary.wiley.com/action/doSearch?ContribAuthorRaw=Desai%2C+Shashwat), [Lynette D. Krebs MPP, MSc](https://onlinelibrary.wiley.com/action/doSearch?ContribAuthorRaw=Krebs%2C+Lynette+D)


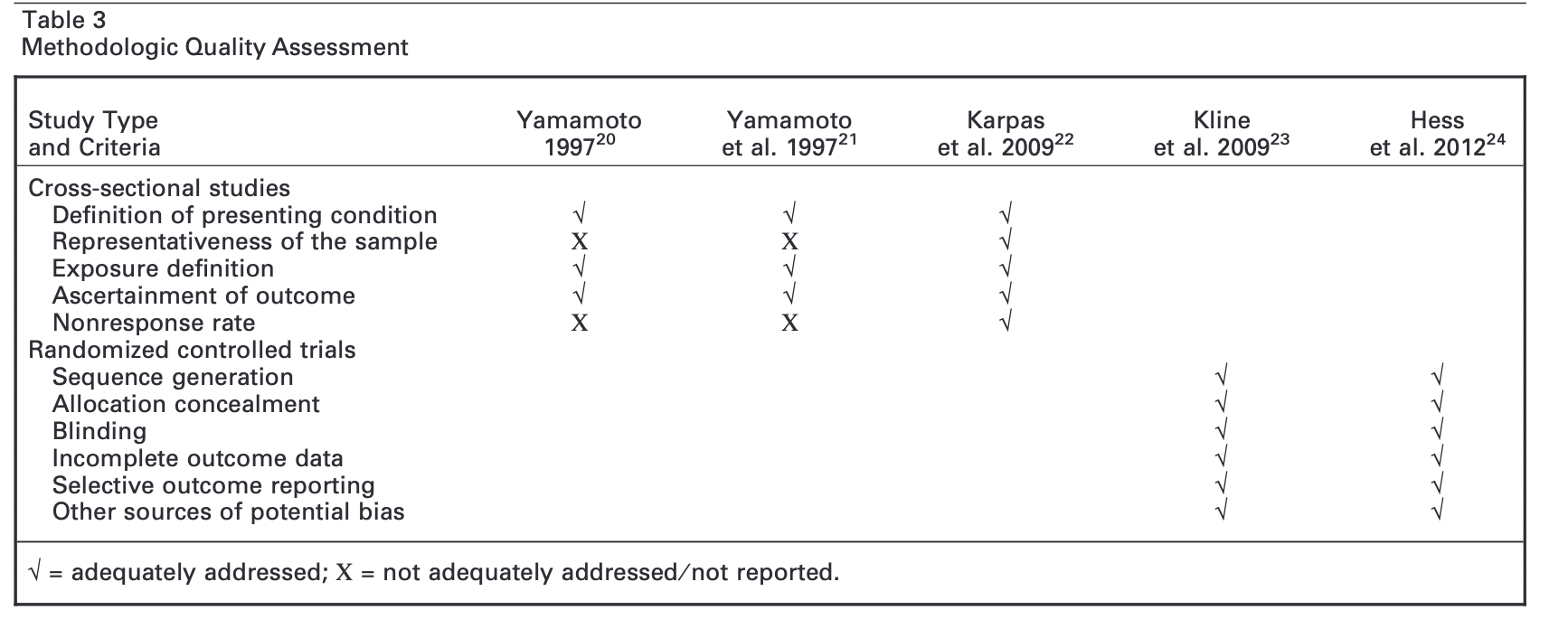


**Supplementary table 19.** Engaging Patients in Health Care Decisionsin the Emergency Department Through Shared Decision-making: A Systematic Review. Darren Flynn, PhD, Only Kline and Hess included in overview.


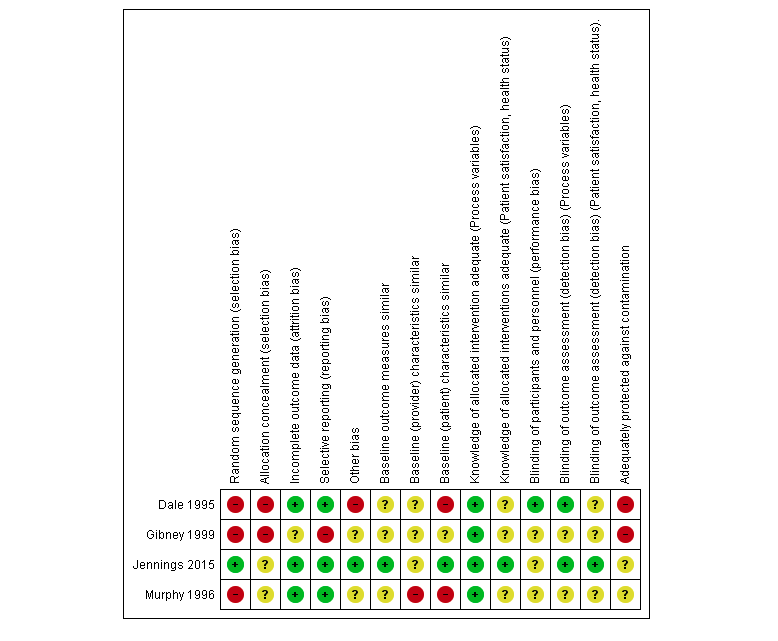


**Supplementary table 20.** Primary care professionals providing non‐urgent care in hospital emergency departments, [Daniela Gonçalves‐Bradley](https://www.cochranelibrary.com/cdsr/doi/10.1002/14651858.CD002097.pub4/information#CD002097-cr-0002). Only Dale and Murphy included in Review.


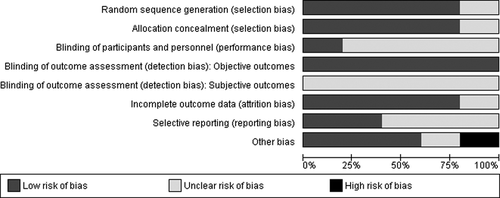


**Supplementary table 21.** Overall, studies were at a moderate risk of bias across outcomes. Taken from Effectiveness and Safety of Short-stay Units in the Emergency Department: A Systematic Review. [James Galipeau PhD](https://onlinelibrary.wiley.com/action/doSearch?ContribAuthorRaw=Galipeau%2C+James)

|  | Goldstein | CT-STAT | ACRIN-PA | ROMICAT II | Median |
| --- | --- | --- | --- | --- | --- |
| **Jadad criteria** |  |  |  |  |  |
| Study described as "randomized?" | 1 | 1 | 1 | 1 | 1 |
| Randomization appropriate? | 1 | 1 | 1 | 1 | 1 |
| Study described as "double blinded?" | 0 | 0 | 0 | 0 | 0 |
| Blinding appropriate? | 0 | 0 | 0 | 0 | 0 |
| Adequate description of withdrawals? | 1 | 1 | 1 | 1 | 1 |
| Statistical methods adequately described? | 1 | 1 | 1 | 1 | 1 |
| Clear description of inclusion and exclusion criteria? | 1 | 1 | 1 | 1 | 1 |
| Method of assessing for adverse effects described? | 1 | 1 | 1 | 1 | 1 |
| Median Jadad Score | 6 | 6 | 6 | 6 | 6 |
|  |  |  |  |  |  |
| **Cochrane Risk of Bias** |  |  |  |  |  |
| Was the allocation sequence adequately generated? | Low | Low | Low | Low | Low |
| Was allocation adequately concealed? | Low | Low | Low | Low | Low |
| Was knowledge of the allocated intervention prevented? | Unclear | Unclear | Unclear | Unclear | Unclear |
| Were incomplete outcome data adequately addressed? | Low | Low | Low | Low | Low |
| Free of other problems that risk bias? | Unclear | Unclear | Low | Low | Unclear |

,

**Supplementary Table 22.** Results of quality of reporting assessment using Jadad scale and risk of bias using the Cochrane tool for assessment of risk of bias. Outcomes After Coronary Computed Tomography Angiography in the Emergency Department: A Systematic Review and Meta-Analysis of Randomized, Controlled Trials**.** [Edward Hulten](https://www.jacc.org/doi/full/10.1016/j.jacc.2012.11.061)


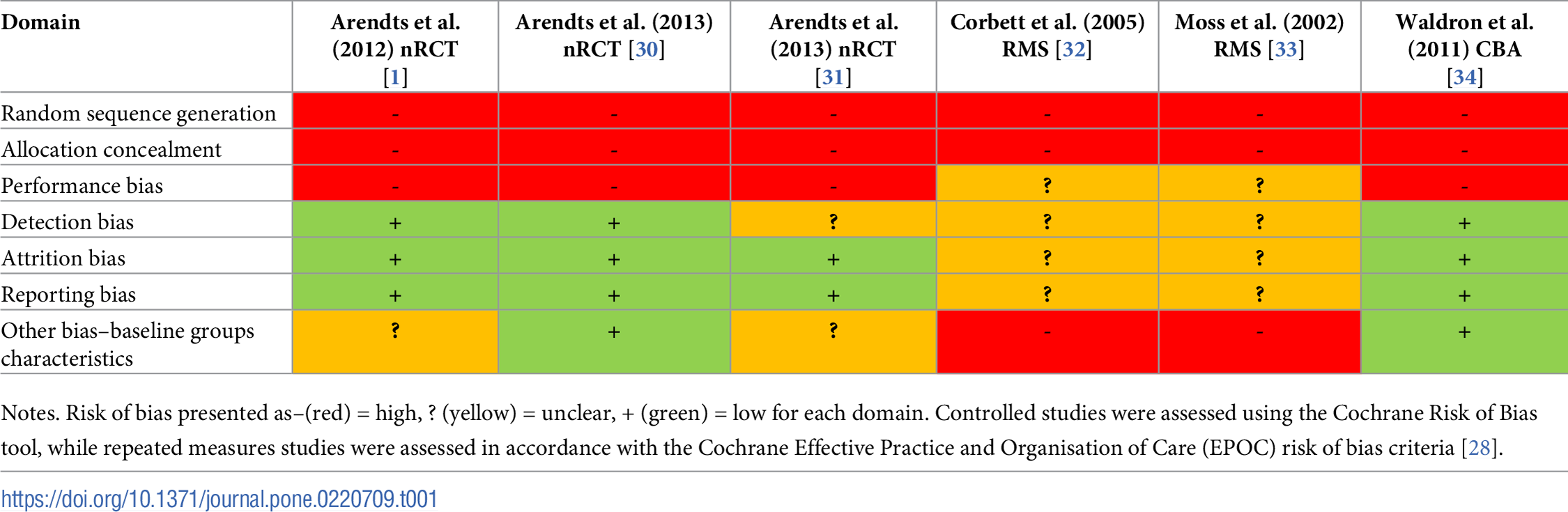


**Supplementary table 23.** Impact of early assessment and intervention by teams involving health and Social Care Professionals in the emergency department: A systematic review; [Marica Cassarino](https://pubmed.ncbi.nlm.nih.gov/?sort=date&size=50&term=Cassarino+M&cauthor_id=31365575) [^1^](https://pubmed.ncbi.nlm.nih.gov/31365575/#affiliation-1), https://doi.org/10.1371/journal.pone.0220709.t001
