# Supplementary material for: Emergency department interventions and their effect on subsequent healthcare resource use after discharge: an overview of systematic reviews
Source: Scand J Trauma Resusc Emerg Med. 2025 May 1;33:76. doi: 10.1186/s13049-025-01377-4 (PMC12044817; doi:10.1186/s13049-025-01377-4)
Supplement: Supplementary file 4 — Additional file 4. [file 13049_2025_1377_MOESM4_ESM.docx]

Testing and cost

A review by Flynn *et al* ^1^ demonstrated that the use of a chest pain decision aid decreased cardiac stress testing at 30 days and pre-test probability reduced thoracic imaging. This is based on high confidence narrative data based on two primary studies with low RoB in adults presenting with chest pain to the ED. ^2,3^

In the frequent attender cohort, interventions including case management, care plans, social work home visits, diversion strategies to non-urgent care, counselling, care co-ordination, pain protocols reduced costs in frequent attenders. Case management was the most common strategy studied and most common intervention to reduce costs. This is based on high confidence data from two reviews by Moe *et al*  ^4^ and Berkman *et al* ^5^ and moderate confidence data by Wong *et al* ^6^.

1. Flynn, D. *et al.* Engaging patients in health care decisions in the emergency department through shared decision-making: a systematic review. *Acad. Emerg. Med. Off. J. Soc. Acad. Emerg. Med.* **19**, 959–967 (2012).

2. Hess, E. P. *et al.* The Chest Pain Choice Decision Aid. *Circ. Cardiovasc. Qual. Outcomes* **5**, 251–259 (2012).

3. Kline, J. A. *et al.* Pretest probability assessment derived from attribute matching. *BMC Med. Inform. Decis. Mak.* **5**, 26 (2005).

4. Moe, J. *et al.* Effectiveness of Interventions to Decrease Emergency Department Visits by Adult Frequent Users: A Systematic Review. *Acad. Emerg. Med.* **24**, 40–52 (2017).

5. Berkman, N. D. *et al.* Management of High-Need, High-Cost Patients: A “Best Fit” Framework Synthesis, Realist Review, and Systematic Review. (2021) doi:10.23970/AHRQEPCCER246.

6. Wong, C. K. *et al.* The Characteristics and Effectiveness of Interventions for Frequent Emergency Department Utilizing Patients With Chronic Noncancer Pain: A Systematic Review. *Acad. Emerg. Med.* **27**, 742–752 (2020).
