# Supplementary material for: Emergency department interventions and their effect on subsequent healthcare resource use after discharge: an overview of systematic reviews
Source: Scand J Trauma Resusc Emerg Med. 2025 May 1;33:76. doi: 10.1186/s13049-025-01377-4 (PMC12044817; doi:10.1186/s13049-025-01377-4)
Supplement: Supplementary file 5 — Additional file 5. [file 13049_2025_1377_MOESM5_ESM.docx]

***Outcome 2c -* Interventions that increased scheduled healthcare resource as their aim**

Primary care follow-up

Interventions (patient navigation for ED patients and emergency room decision-support (ERDS) program) designed for ED frequent attenders to seek more ‘appropriate’ healthcare options other than the ED, resulted in increased primary care visits as intended. This is based on high confidence data from Berkman *et al* ^1^. The GRADE was low and evidence was based on one RCT with favourable findings (RoB = some concerns) and one observational study with favourable findings (RoB= some concerns).

In patients presenting to the ED with asthma, a review by Villa-Roel *et al* ^2^, found educational interventions increased follow-up rates with a primary care practitioner as intended. Interventions were: arranged follow-up, follow-up phone calls, faxed letters, asthma action plans and transportation vouchers plus oral steroids. The risk ratio compared to usual care was 1.6 (95% CI = 1.31 to 1.87), indicating the increased likelihood of planned follow-up. Grade was not reported, and results were based on five RCTs with a predominantly low RoB.

Care transition interventions improved the rate of follow-up with primary care or specialist providers in adult ED patients. Care transition interventions were defined as educational support (face-to-face, video-based or telephonic), reminders (mailed, text or telephonic), appointment scheduling and ED-based discharge instructions and case management programs. The odds of outpatient follow-up rate increased versus usual care (OR 1.79, 95% CI 1.43,2.24). This is based on high confidence meta-analysis data from Aghajafari *et al* ^3^, which included 20 studies (n=8178 patients), the GRADE outcome was low.

Outpatient/specialist follow-up

Case management interventions in ED frequent attenders increased outpatient visits as intended. This is based on high confidence data from Althaus *et al* ^4^, which analysed data from two primary studies (﻿Quality Criteria for NCBA studies = "Y=6 U=2 N=2, Y=7 U=1 N=2").

Data from a high confidence review by Moe *et al* ^5^ reported the effect of interventions on frequent attenders and their impact on outpatient resource use. No detail was provided on whether interventions aimed to increase or decrease outpatient use. Six studies reported interventions (three of which listed case management interventions, one listed care plans and two diversion strategies to non-urgent care) that increased outpatient attendances. Four studies had a moderate RoB and two a high RoB. GRADE was not reported. Printout case notes and case management interventions, from two further studies with high RoB, had no effect on outpatient resource use. One study, with a moderate RoB, used a social work home visit intervention and outpatient attendances.

Multi-disciplinary team protocols aimed at decreasing imaging for lower back pain, increased the use of physiotherapy and rehabilitation services as planned. This is based on moderate confidence narrative data from Liu *et al* ^6^ based on one study with low BAQA scores.

1. Berkman, N. D. *et al.* Management of High-Need, High-Cost Patients: A “Best Fit” Framework Synthesis, Realist Review, and Systematic Review. (2021) doi:10.23970/AHRQEPCCER246.

2. Villa-Roel, C. *et al.* Effectiveness of Educational Interventions to Increase Primary Care Follow-up for Adults Seen in the Emergency Department for Acute Asthma: A Systematic Review and Meta-analysis. *Acad. Emerg. Med. Off. J. Soc. Acad. Emerg. Med.* **23**, 5–13 (2016).

3. Aghajafari, F., Sayed, S., Emami, N., Lang, E. & Abraham, J. Optimizing emergency department care transitions to outpatient settings: A systematic review and meta-analysis. *Am. J. Emerg. Med.* **38**, 2667–2680 (2020).

4. Althaus, F. *et al.* Effectiveness of interventions targeting frequent users of emergency departments: a systematic review. *Ann. Emerg. Med.* **58**, 41-52.e42 (2011).

5. Moe, J. *et al.* Effectiveness of Interventions to Decrease Emergency Department Visits by Adult Frequent Users: A Systematic Review. *Acad. Emerg. Med.* **24**, 40–52 (2017).

6. Liu, C. *et al.* Effectiveness of Interventions to Decrease Image Ordering for Low Back Pain Presentations in the Emergency Department: A Systematic Review. *Acad. Emerg. Med.* **25**, 614–626 (2018).
